# Ammonium metabolism rewiring in the prostate cancer microenvironment: Mechanisms and clinical prospects

**DOI:** 10.3389/fonc.2025.1673513

**Published:** 2025-10-16

**Authors:** Zihao Ye, Hao Wu, Zhanhao Li, Ruizhe Ye, Yingliang Rao, Bin Liu, Baoshan Gao

**Affiliations:** ^1^ Department of Urology II, The First Hospital of Jilin University, Changchun, China; ^2^ School of Basic Medicine, Tongji Medical College, Huazhong University of Science and Technology, Wuhan, China; ^3^ Department of Urology, China-Japan Hospital of Jilin University, Changchun, China

**Keywords:** ammonium metabolism, prostate cancer, ADT, TME, SLC

## Abstract

Ammonium metabolism represents a critically understudied yet pivotal driver of prostate tumorigenesis and tumor microenvironment (TME) remodeling. The interplay between tumor metabolic reprogramming and the tumor microenvironment has emerged as a critical frontier in oncology research. While previous studies on prostate cancer metabolism have predominantly focused on lipid metabolism and the Warburg effect, the role of ammonium metabolism, particularly the urea cycle in tumor immune regulation remains insufficiently explored. This metabolic reprogramming constitutes a central node connecting catabolic nutrient breakdown to anabolic biosynthesis by integrating upstream amino acid deamination and transamination reactions with downstream pathways, generating key intermediates including α-ketoglutarate, coenzyme A, and citrate that concurrently fuel the tricarboxylic acid cycle and macromolecular synthesis. Crucially, oncogenic drivers such as Myc and p53 modulate this flux through epigenetic regulation of core enzymes such as glutaminase, glutamine synthetase and ornithine transcarbamylase, thereby channeling metabolism toward tumor progression. The immunomodulatory consequences manifest through dual mechanisms including TME immunosuppression driven by M2 macrophage polarization and immune evasion mediated via glutathione dependent redox homeostasis disruption. Beyond its established role in modulating redox homeostasis, ammonium metabolic reprogramming may additionally trigger novel cell death modalities such as ferroptosis by GSH/GPX4 axis. This emerging pathway offers promising therapeutic avenues for prostate cancer intervention. Synthesizing mechanistically validated insights from *in vivo* or *in vitro* models and clinical trials of ammonium-targeting inhibitors, this review proposes novel therapeutic strategies and candidate biomarkers. Moreover, the unique citrate and polyamine metabolism characteristics of prostate cancer may be impacted by these processes, offering promising avenues for future treatments.

## 1 Introduction

Prostate cancer (PCa) is the second most common solid tumor in men worldwide and the fifth leading cause of cancer-related death. Its incidence and mortality rates vary by region (1). In 2020, there were more than 1.4 million new cases and more than 375,000 deaths globally. Prostate cancer can be classified into androgen-sensitive and androgen-insensitive types, which influence treatment options. Common treatments include active surveillance, chemotherapy, radiotherapy, hormone therapy, surgery, and focal therapy (2). Surgery and radiotherapy are effective for localized prostate cancer, whereas androgen deprivation therapy is the standard treatment for advanced or metastatic cases (3). Although 80% of prostate cancer cases are localized, 20% to 40% of patients experience recurrence within five years, and approximately 20% of localized cases progress to metastatic hormone-sensitive prostate cancer (mHSPC), which can further evolve into metastatic castration-resistant prostate cancer (mCRPC) (4). Therefore, there is an urgent need to develop new treatment strategies, especially for CRPC.

Metabolic reprogramming, characterized by alterations in lipid, glucose, and amino acid metabolism, is a hallmark of cancer and enables malignant cells to adapt to the unique features of the tumor microenvironment (TME) (5). In prostate cancer, a shift from lipid-dominated to glucose-dependent metabolism occurs during disease progression, particularly from early to metastatic stages, which may contribute to therapeutic resistance. Metastatic prostate cancer uses glycolysis to maintain an acidic tumor microenvironment, which suppresses immune cell activity and promotes immune evasion (6, 7). In bone metastasis, accompanied with adipocyte, prostate cancer cells exhibit high glycolytic rates and increased HIF-1α expression, which regulates Warburg effect genes, leading to enhanced lactate production and inhibition of oxidative phosphorylation (8). These findings confirm that glycolysis is a crucial metabolic pathway in prostate cancer metastasis and progression. These metabolic changes may lead to drug resistance in cancer cells, enabling them to evade therapeutic treatments (9). While extensive studies have explored lipid and glucose pathways, limited therapeutic opportunities have emerged due to metabolic heterogeneity and redundancy (10, 11). Specifically, in prostate cancer, PI3K activation and MYC-induced lipid metabolism promote glycolysis and contribute to metabolic heterogeneity (12). Additionally, FASN inhibitors can be bypassed by exogenous fatty acids, and cancer cells regulate acetyl-CoA sources like acetate and glutamine to adapt to metabolic stress (13, 14). Recently, ammonium metabolism, a key node integrating amino acid turnover, nitrogen balance, and intracellular pH regulation, has attracted growing interest (15, 16). Under nutrient-deprived conditions, Intermediates such as glutamine and glutamate not only serve as nitrogen and carbon sources for anabolic processes but also participate in TME remodeling and immunoregulation, particularly under nutrient deprivation, relevant to prostate cancer biology (17).

This review summarizes recent studies on the role of ammonium metabolism in the prostate cancer microenvironment, discusses its potential as a therapeutic target, and explores its application as a diagnostic biomarker for prostate cancer to improve both detection and prognosis.

## 2 Ammonium metabolism in tumor cells: sources, fates, and functional implications

### 2.1 Metabolic reprogram and crosstalk of ammonium metabolism in prostate cancers

PCa cells exhibit distinct ammonium metabolism characterized by dysregulated upstream amino acid catabolism and coordinated crosstalk with glucose and lipid metabolic pathways. Accumulating evidence establishes asparagine synthetase (ASNS) as a critical driver of malignant progression in solid tumors including prostate cancer, where it sustains tumorigenesis through dual metabolic reprogramming (18–21). In CRPC, TP53 mutation or deletion drives ASNS upregulation via the mTOR/ATF4 axis, enhancing cellular reliance on ASNS-mediated asparagine biosynthesis. This metabolic plasticity enables tumor survival in androgen-deprived microenvironments (22). This regulation typically arises due to the disruption of the balance between the recruited corepressor and coactivator following TP53 gene mutation. Another mechanism involves the mutant CRCP of TP53 predominantly binding to ATF4, thereby exerting an inhibitory effect, in contrast to the wild-type CRPC of TP53. TP53 mutation transcriptionally suppresses ASNS expression, thereby disrupting asparagine-aspartate homeostasis. Crucially, this regulatory circuit operates bidirectionally, asparagine and aspartate reciprocally modulate AMPK-mediated p53 activation through allosteric binding to LKB1 that bidirectionally regulates its kinase activity (23). Recent studies reveal ASNS gene amplification with concomitant mRNA overexpression, augmenting asparagine production (19). Aspartate fuels tumor progression by integrating into glycolysis and lipogenesis pathways while suppressing apoptosis under glucose deprivation via JNK/SAPK activation (24). Previous studies have suggested that TP53-mutant CRPC exhibits metabolic vulnerability as an adaptive response to the nutrient-deprived tumor microenvironment (23). Therapeutic strategies targeting asparagine metabolism have shown promising efficacy in this context.

In PCa, glutamine serves as a critical upstream hub in ammonium metabolism, with tumors exhibiting profound metabolic rewiring to sustain proliferative and stress-adaptive demands. Glutamine metabolism serves as a metabolic intersection in prostate cancer ammonium reprogramming, where this conditionally essential amino acid propels tumor proliferation through multifaceted biosynthetic and bioenergetic contributions including its catabolic flux furnishes precursors for *de novo* purine/pyrimidine biosynthesis and hexosamine pathway activation, drives reductive carboxylation-dependent lipogenesis, sustains redox homeostasis via glutathione synthesis and NADPH regeneration, generates non-essential amino acids, and fuels mitochondrial oxidative phosphorylation through α-KG (α-ketoglutarate) mediated anaplerosis, collectively establishing glutaminolysis as an indispensable axis supporting prostate cancer malignancy (9, 25). Under hypoxia, PCa cells divert excess nitrogen, derived from upstream amino acids and accumulated ammonium, toward dihydroorotate synthesis rather than uridine monophosphate (UMP) production, effectively circumventing ammonium toxicity (26). Mechanistically, hypoxia-induced NADH accumulation, rather than canonical HIF1 signaling, drives this glutamine metabolic reprogramming (27). Unlike normal cells, which utilize glutamine for basal nitrogen/energy homeostasis, PCa cells elevate glutamine uptake and catabolism to meet biosynthetic demands (28, 29).Glutamine-derived carbon undergoes reductive carboxylation to generate acetyl-CoA for fatty acid synthesis, while its nitrogen is channeled into nucleotide biosynthesis (30). Concurrently, glutamine metabolism sustains redox balance via glutathione synthesis, enabling adaptation to oxidative stress in hypoxic, nutrient-deprived microenvironments (31). This metabolic dependency positions glutaminolysis as a therapeutic strategy. From the perspective of signaling pathways, mainstream research indicates that glutamine addiction is associated with the progression and metastasis of prostate cancer cells through three distinct pathways: the AR pathway, the MYC pathway, and the PTEN/PI3K/mTOR pathway. ASCT2 (SLC1A5, solute carrier transporters) is a critical transporter responsible for glutamine uptake in prostate cancer cells with its expression is elevated in tumor tissues and further increased in CRPC. ASCT2 is directly regulated by AR signaling (32). In AR-sensitive cells such as LNCaP, androgens like dihydrotestosterone (DHT) significantly upregulate ASCT2 expression and enhance glutamine uptake. In contrast, AR-insensitive cell lines (e.g., DU-145, PC-3) also rely on ASCT2-mediated glutamine transport but are not directly modulated by AR signaling (33, 34). GLS, the rate-limiting enzyme in glutamine catabolism, exhibits markedly higher expression in AR-insensitive cells, particularly DU-145, contributing to their increased glutamine dependency. Inhibition of GLS, using agents such as BPTES or siRNA, effectively reduces viability across all PCa cell lines, with AR-independent cells showing heightened sensitivity (35). From a therapeutic perspective, combined inhibition of AR (androgen receptor) signaling and GLS activity yields synergistic anti-tumor effects in AR-sensitive cells. In contrast, targeting glutamine metabolism alone, especially through GLS inhibition, represents a more effective strategy for AR-independent tumors. These findings support a tailored therapeutic approach based on the AR status of prostate cancer.

MYC functions as a central regulator of glutamine metabolism in prostate cancer by repressing miR-23a/b, thereby relieving the inhibition of mitochondrial GLS and promoting glutaminolysis, particularly in androgen-independent PC-3 cells (36, 37). The antitumor efficacy of the GLS inhibitor CB-839 is strongly MYC-dependent (38). In AR-positive cells such as LNCaP, MYC also upregulates ASCT2 expression and enhances glutamine uptake in an androgen-responsive manner (39). The metabolic role of MYC is further modulated by PTEN/PI3K signaling, where concurrent mTORC1 activation in PTEN-deficient cells amplifies glutamine metabolism (40). Glutamine metabolic reprogramming not only sustains tumor proliferation but also contributes to therapy resistance; inhibition of GLS or MYC sensitizes prostate cancer cells to radiotherapy, while autophagy enables glutamine-independent cells to withstand metabolic stress. Additional regulatory mechanisms include SRC-2–mediated reductive carboxylation, compensatory glutamine addiction induced by PDHA1 loss, Guanine Monophosphate Synthetase(GMPS) driven purine biosynthesis, reciprocal regulation between GLS and CAD, epigenetic modulation via MeCP2/DNMT, and C5a complement signaling that promotes glutamine consumption in castration-resistant prostate cancer (41–43). Notably, PC-3M (prostate cancer cell lines) subpopulations with stem-like features exhibit heightened glutamine dependency characterized by elevated GLS1 expression and enhanced reductive carboxylation activity, forming a bidirectional regulatory loop between glutamine metabolism and epigenetic reprogramming (44). Collectively, these findings underscore the essential role of glutamine metabolism in prostate cancer progression and therapy resistance, and support the development of targeted combinatorial strategies.

**Figure 1:**
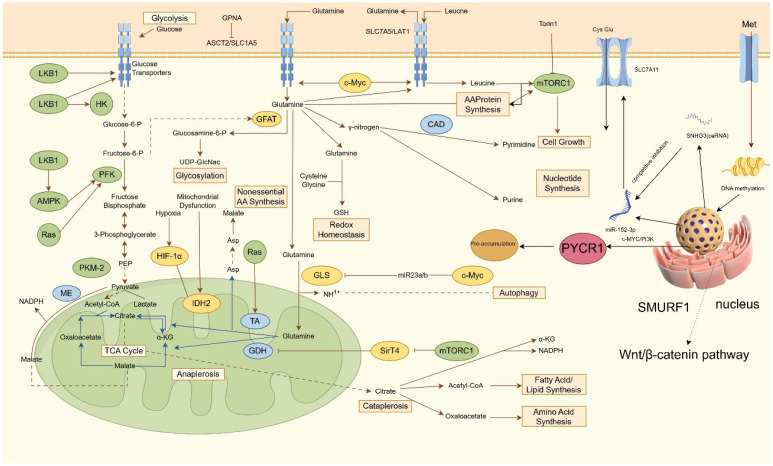
Regulatory mechanism of ammonium metabolism in PCa. This diagram outlines a coordinated network of cellular nutrient metabolism and signaling pathways that support growth and survival. Glucose enters via transporters and undergoes glycolysis, regulated by HK, PFK, and the LKB1-AMPK-Ras axis, yielding pyruvate. Amino acid transporters ASCT2 and SLC7A5 import glutamine and leucine; leucine activates mTORC1 to promote protein synthesis. Glutamine metabolism diverges into multiple pathways: it supports hexosamine biosynthesis (via GFAT), nucleotide synthesis (via CAD for pyrimidines), non-essential amino acid production, and glutathione-mediated redox balance. Converted to glutamate by GLS, it also fuels the TCA cycle, where enzymes including IDH2 and GDH sustain anaplerosis and intermediate turnover, influenced under hypoxia by HIF-1α. Signaling through mTORC1, SirT4, and the Wnt/β-catenin-SMURF1 pathway regulates gene expression and epigenetic modifications. Additionally, non-coding RNAs such as miR-152-3p and SNHG3 fine-tune processes including autophagy and proline metabolism via targets like c-Myc/PI3K, ensuring integrated control of metabolic and proliferative functions. HK, hexokinase; PFK, phosphofructokinase, UDP-GlcNac, Uridine Diphosphate N-Acetylglucosamine; GSH, glutathione; SNHG3, Small Nucleolar RNA Host Gene; ceRNA, competing endogenous RNAs.

Metabolomic profiling reveals elevated alanine levels in PCa versus normal prostate tissues, potentially reflecting heightened membrane biosynthesis requirements (45, 46). Alanine transamination supports glutamate oxidation to α-KG, providing an alternative lipogenic carbon source distinct from pyruvate/lactate flux, while excess alanine may directly fuel protein synthesis (47, 48). Studies in PCa suggest that arginine derived metabolites promote tumor proliferation by suppressing apoptosis through redox modulation (49). Mechanistically, nitric oxide synthase (NOS) converts arginine, NADPH, and oxygen into nitric oxide, which inhibits apoptosis by altering cellular redox states (50, 51). PCa cells exhibit upregulated tryptophan-hydroxylating enzymes and aromatic L-amino acid decarboxylases, key drivers of tryptophan metabolic reprogramming. These enzymes orchestrate tryptophan catabolism, with tryptophan hydroxylase (TPH) catalyzing the conversion of tryptophan (Trp) to serotonin. Elevated expression of tryptophan-2,3-dioxygenase 2 (TDO2) and indoleamine 2,3-dioxygenase 1 (IDO1) enhances kynurenine (Kyn) production, which fosters PCa progression through tumor-associated immunosuppression and direct pro-survival signaling (52–54). Methionine addiction characterizes PCa ammonium metabolism, with SNHG3 overexpression identified as a key upstream regulator through its interaction with the miR-152-3p/SLC7A11 axis (55, 56). Functional validation confirms SLC7A11 as a direct miR-152-3p target whose overexpression rescues methionine dependency in SNHG3-deficient PCa cells, establishing the SNHG3/miR-152-3p/SLC7A11 regulatory axis (**Figure 1**). Proline metabolism, regulated by oncogenic c-MYC and PI3K pathways, modulates AR transcriptional activity through cyclin D3/CDK11p58-mediated serine phosphorylation, a mechanism requiring further mechanistic exploration (37, 55, 57, 58).

**Figure 2:**
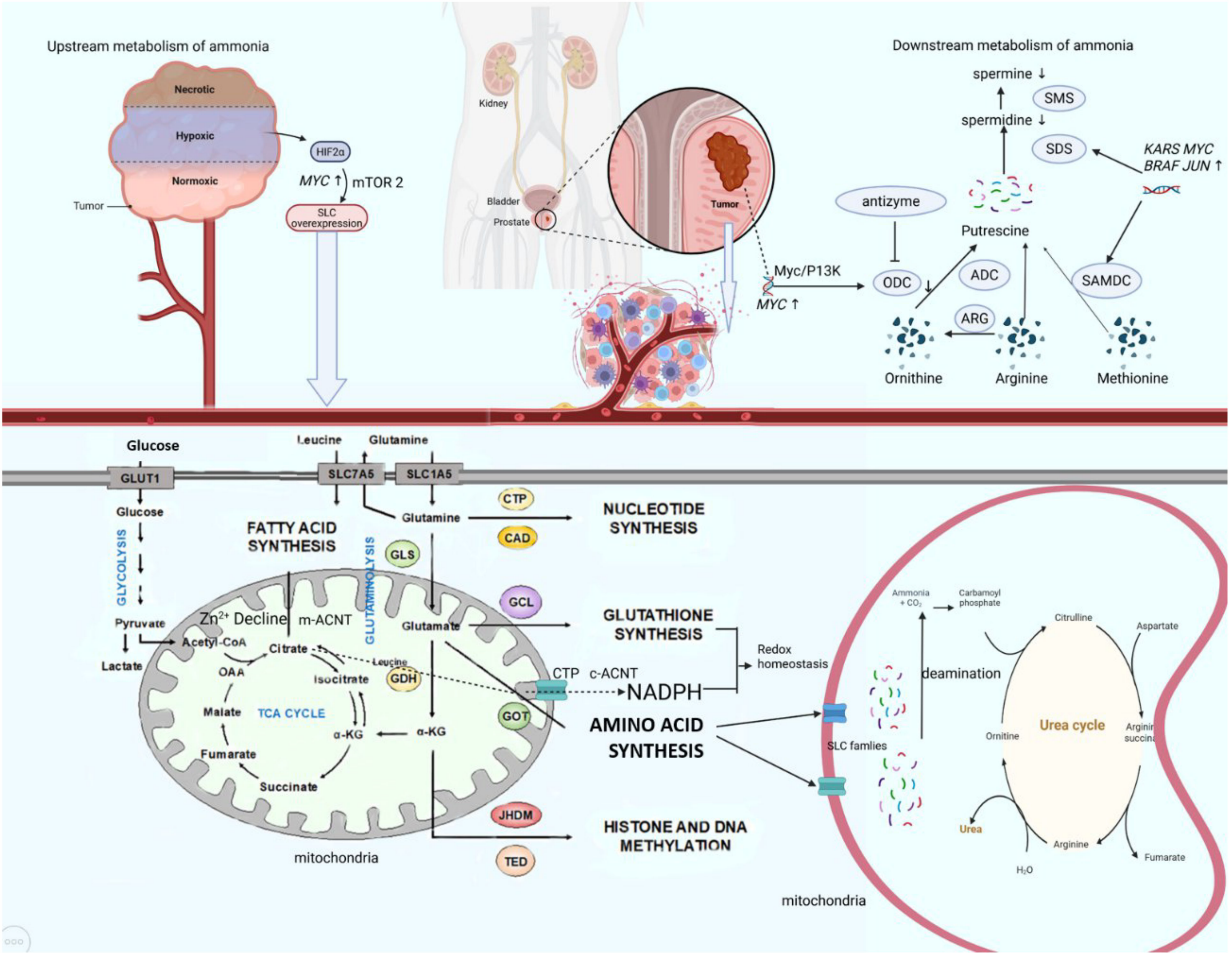
Ammonium metabolic reprogram axis in PCa. In the tumor microenvironment, hypoxia activates HIF-2α, which upregulates MYC and subsequently enhances mTORC2 signaling to promote expression of SLC transporters. This leads to increased uptake of glucose and amino acids such as glutamine. Glutamine is converted to glutamate by GLS, supporting biosynthesis of lipids, nucleotides, and glutathione. Concurrently, the ornithine–arginine–methionine pathway drives polyamine synthesis via ODC and SAMDC, regulated by MYC, JUN, and BRAF. The urea cycle mitigates ammonia toxicity. Oncogene activation disrupts these interconnected metabolic networks, sustaining tumor growth. HIF, hypoxia inducible factor; GLS, glutaminase; CTP, cytidine triphosphate; CAD, Caspase-activated deoxyribonulease; ACNT, aconitase; ODC, ornithine decarboxylase; ARG, arginase; ADC, arginine decarboxylase; SMS, spermine synthase; SDS, spermidine synthase.

The downstream metabolites of ammonium metabolism in prostate cancer cells, notably citrate and polyamines, exert significant functional influences on prostate cancer development. The prostate gland contains the highest polyamine concentrations in the human body, with spermine constituting the predominant polyamine species. Androgens coordinately induce the enzymatic activities of key polyamine biosynthetic enzymes, ornithine decarboxylase (ODC), S-adenosylmethionine decarboxylase (SAMDC), and spermidine synthase (SDS), with predominant expression localized to prostatic glandular epithelial cells (59–61). Murine ODC gene studies reveal an androgen response element-like sequence within the ODC promoter that binds androgen receptor *in vitro*. The functions of ODC and polyamines in prostate tissue are mechanistically linked to cellular proliferation and secretory activity (62). Recent investigations demonstrate overexpression of ODC, the rate-limiting enzyme in polyamine metabolism, in prostate cancer cells, accompanied by elevated ODC protein and mRNA levels (63, 64). Comparative analyses of polyamine levels in human normal, benign, and malignant prostatic tissues reveal elevated spermine concentrations in normal and benign hyperplastic prostates, contrasting with reduced spermine levels in tumor tissues, particularly metastatic prostate cancer (65). This marked depletion of prostatic spermine may signify the phenotypic transition from benign to malignant states. Consequently, ODC has been proposed as a proto-oncogene expression product in prostate carcinogenesis. Notably, enzymes governing polyamine biosynthesis, including glutamic oxaloacetic transaminase 2 (GOT2), aminoacylase-1 (ACY1), ODC, and SDS, exhibit overexpression in prostate cancer, while ornithine aminotransferase (OAT) demonstrates insufficient expression (66). The net effect redirects substrate flux toward polyamine biosynthesis while diverting it from proline synthesis, potentially contributing to oncogenic transformation. Emerging evidence suggests these elevated enzymatic levels are sustained through combinatorial mechanisms: enhanced biosynthesis, increased transporter activity, and reduced catabolism. Multiple oncogenes, including *MYC*, *JUN*, *FOS*, *KRAS*, and *BRAF*, are associated with ODC and SAMDC expression, with ODC and SAMDC being particularly linked to *MYC* activation (67, 68) (**Figure 2**).

Mechanistically, PCa cells exhibit impaired ODC antizyme regulation (69, 70). In spermine-insensitive cells, antizyme levels fail to upregulate, resulting in unabated ODC activity due to insufficient inhibition and degradation. Reduced antizyme levels observed across multiple cancer types suggest tumor-suppressive properties of ODC antizyme. Paradoxically, while most cancers demonstrate elevated intracellular polyamines correlating with proliferative metabolism, prostate cancer exhibits an inverse polyamine profile, warranting further mechanistic investigation. The prostate gland exhibits unique metabolic features characterized by extraordinary citrate production (up to 180 mM in prostatic fluid) to support sperm energetics, sustained through zinc-mediated inhibition of mitochondrial aconitase(m-ACNT), which blocks citrate-to-isocitrate conversion (71). In prostatic cells, citrate within the mitochondrial TCA (tricarboxylic acid) cycle undergoes compartment-specific metabolic partitioning. Mitochondrial citrate is either exported to the cytosol via solute carrier family 25 transporters, notably the citrate transport protein (CTP) embedded in the inner mitochondrial membrane, to fuel cytosolic fatty acid and sterol biosynthesis, or retained intramitochondrially to contribute to ATP generation (72). Additionally, extracellular citrate derived from systemic circulation can be imported into prostatic cells through SLC13 family transporters, a class of citrate carriers ubiquitously expressed across multiple organ systems (73, 74). This dual citrate sourcing, endogenous mitochondrial production and exogenous uptake, underscores the metabolic adaptability of prostatic cells in maintaining citrate homeostasis for both bioenergetic and biosynthetic demands. In PCa, metabolic reprogramming disrupts this homeostasis through sequential and interconnected mechanisms. First, dramatic Zn²^+^ depletion relieves m-ACNT suppression, enabling mitochondrial citrate catabolism. Concurrently, a metabolic shift to “citrate oxidation” splits citrate into acetyl-CoA, fueling lipogenesis via overexpressed fatty acid synthase (FAS), and α-KG which sustains TCA cycle activity to support proliferation. Despite compensatory upregulation of sodium-dependent citrate transporters (NaCitT) in advanced tumors, accelerated citrate consumption consistently outpaces extracellular uptake (75). Furthermore, elevated cytosolic aconitase (c-ACNT) and isocitrate dehydrogenase (ICD2), synergistically enhanced by iron accumulation, drive citrate flux toward NADPH and α-KG generation, thereby exacerbating intracellular citrate depletion. Paradoxically, while normal prostate physiology prioritizes polyamine synthesis (e.g., spermine), regulated by androgen-dependent enzymes like ODC to support secretory functions, PCa progression exhibits a contradictory polyamine landscape: spermine levels decline sharply despite ODC overexpression, a phenomenon contrasting with polyamine accumulation patterns in other cancers. Collectively, this dual metabolic identity, preserving physiological specialization in citrate production while hijacking polyamine and citrate pathways for oncogenic rewiring, not only distinguishes PCa from other malignancies but also unveils targetable vulnerabilities for diagnostic and therapeutic innovation.

### 2.2 Metabolic reprogramming of ammonium metabolism as pivotal part of tumor metabolism

Ammonium metabolism in tumor cells exhibits distinct features compared to normal cells, serving dual roles in energy production and substrate provision for proliferation. Similar to normal cells, tumor cells generate ammonium primarily via amino acid deamination, notably through the glutamine cycle, where glutamate dehydrogenase (GDH) catalyzes glutamate deamination to produce free ammonium and α-KG. GDH produces α-KG and ammonia through oxidative deamination, while transaminases convert α-KG into glutamate, enabling flexible regulation of nitrogen metabolism (76). Further studies indicate that glutamine serves as both a nitrogen and carbon source in prostate cancer, with higher expression of glutamine transporters (ASCT2), GDH1, AST1, and GLUL (Glutamine synthetase). This elevated enzyme expression is linked to tumor metabolic reprogramming and increased glutamine dependency, emphasizing the central role of glutamine metabolism in ammonia metabolism in prostate cancer (77, 78). In normal physiology, ammonium derived from deamination is transported as glutamine or alanine to hepatocyte mitochondria, where it undergoes enzymatic conversion to urea via the urea cycle (Details in **Table 1**). This cycle involves sequential reactions that ultimately produce urea, which is excreted renally to prevent neurotoxic ammonium accumulation. To meet heightened metabolic demands, tumor cells exhibit elevated glutamine consumption. Animal studies reveal that colorectal cancer (CRC) cells accumulate ammonium due to downregulation of transcription factors HNF4-α and ornithine transcarbamylase (OTC), a phenomenon also observed in CRC patients. Concurrent overexpression of SLC4A11, glutaminase (GLS), and GDH further amplifies glutamine derived ammonium production (79). Intriguingly, tumor-associated hyperammonemia (elevated glutamate and NH4+) promotes lipogenesis via glucose-dependent mechanisms. Specifically, ammonium stimulates sterol regulatory element-binding protein (SREBP) cleavage, enhancing nuclear translocation and upregulating fatty acid synthase (FASN) and stearoyl-CoA desaturase 1(SCD1). Conversely, glucose deprivation suppresses SCAP (SREBP cleavage-activating protein) N-glycosylation, blocking SREBP activation, suggesting synergistic regulation of lipogenesis by glucose and ammonium (80). Beyond energy metabolism, glutamine-derived ammonium contributes to nucleotide synthesis, though its downstream nitrogen utilization pathways remain poorly defined (81, 82). In estrogen receptor (ER)-positive breast cancer, Carbamoyl Phosphate Synthetase 1(CPS 1) (normally absent in hepatic cells), GS (glutamine synthetase), and GDH are overexpressed. Isotopic tracing with 15N-labeled glutamine revealed that 57% of ammonium is recycled via GDH-mediated reductive amination, generating 15N-labeled amino acids (excluding proline and glutathione, which are synthesized directly from glutamate) (83). Spinelli et al. demonstrated that tumors produce ammonia through glutamine metabolism, which is converted into glutamate and downstream amino acids via reductive amination to support cellular nitrogen requirements. Using 15N-labeled glutamine and HILIC-MS, the study tracked ammonia metabolism, revealing its recycling in tumor cells into glutamate and its derivatives. Although conducted in breast cancer cell lines, recent studies have confirmed similar metabolic processes in prostate cancer (77).These findings highlight tumor-specific adaptations in ammonium metabolism, emphasizing its role in sustaining proliferation and metabolic flexibility. Aside from glutamine, proline is also a key type of amino acid which is active in cancer cells. Proline metabolism not only supports collagen synthesis for the extracellular matrix (ECM), but also enables tumor cells to recycle collagen-derived proline under nutrient stress in the tumor microenvironment, promoting tumor growth (84, 85). Key enzymes such as P4H are activated by HIF-α (Hypoxia inducible factor-α), enhancing collagen deposition and tumor invasiveness (86, 87).

**Table 1:** The impact of ammonium accumulation in tumor cells.

Cancer type	Material	Ammonium concentration	indicator	Tumor cell growth, invasion and metastases	Reference
Breast cancer	MCF-7	0–20 mM NH4CL in medium	cell growth	proliferate most rapidly at concentrations of 2,3 or 5mM ammonium	(285)
Colorectal Cancer	MC38(C57BL/6J)	0–2 mM in Mice	Tumor weight	tumor volume of mice was the largest at a concentration of 2 mM ammonium	(79)
NSCLC	H1299	0–10 mM NH4CL in medium	lipid synthesis gene	ammonium concentration leads to expression of lipid gene FASN/SCD1	(80)
HCC	HepG2/HUH7	0/10 mM NH4CL in medium	Tumor Volume	The number and diameter of liver spheres observed at a concentration of 10 mM NH_4_Cl were significantly greater than those in the control group	(286)
Breast cancer	HCC1806/4T1/E0771/EMT6	0-0.25 mM NH4/NH3 in medium	cytotoxicity	Under high concentrations of ammonium, the cytotoxic activity of T cells and NK cells is markedly diminished.	(287)
Lymphoma	Raji/Ramos	0–3 mM NH4/NH3 in medium	cytotoxicity		
MM	H929/RPMI8226	0–4 mM NH4/NH3 in medium	cytotoxicity		
Prostate cancer	DU-145/PC3	0–10 mM NH4CL in medium	cell growth	As the ammonium concentration increases, the cell count of prostate cancer cells reaches its peak between 4 and 6 days.	(288, 289)

NSCLC, non-small cell lung cancer; HCC, Hepatocellular carcinoma; MM, Multiple myeloma MC38(C57BL/6J), Tumor-bearing mice inoculated with MC38.

Emerging evidence highlights the oncogenic rewiring of glutamine metabolism through multifaceted mechanisms. The Myc oncoprotein directly stimulates glutamine uptake by binding to promoters of glutamine metabolism genes (e.g., the glutamine transporter SLC1A5) and indirectly enhances glutaminolysis by repressing microRNA miR-23a/b, a negative regulator of GLS1 (36, 88). Conversely, the tumor suppressor p53 upregulates the glutaminase isoform GLS2 (89). Moreover, the transcription factor c-Jun, encoded by the proto-oncogene *JUN*, upregulates GLS through coordinated transcriptional and post-translational mechanisms. Directly, c-Jun binds to the GLS promoter at a v-Jun-homologous response element, enhancing GLS transcription. Indirectly, oncogenic Rho GTPase signaling activates JNK (c-Jun N-terminal kinase), which phosphorylates and stabilizes c-Jun, forming a coherent JNK/c–Jun/GLS promoter axis that amplifies GLS expression and promotes glutamine metabolism in breast cancer cells (90). Additional oncogenic drivers, including IDH1/2 mutations, *STAT1*, *ERK*, and *KRAS*, further modulate glutamine metabolic abundance (91–94). However, current studies on oncogene-driven metabolic reprogramming remain disproportionately focused on carbohydrates and lipids, with limited exploration of ammonium metabolism, a critical gap warranting systematic investigation.

The tumor microenvironment exerts profound bidirectional crosstalk with cancer cell metabolism. Composed of tumor cells, stromal cells, immune populations, and bioactive molecules, the TME imposes nutrient constraints that drive adaptive metabolic responses. HIF-1 orchestrates this adaptation under low oxygen tension, transcriptionally activating glycolysis-associated genes such as glucose transporters (GLUT1/3), glycolytic enzymes (HK1/2, ENO1, PGK1, PKM2), and lactate dehydrogenase A (LDHA), thereby shifting energy production from oxidative phosphorylation to aerobic glycolysis (94–96). This metabolic shift elevates cytosolic lactate concentrations from physiological levels (1.5–3 mM) to pathological levels (10–30 mM) in cancer cells. Proton-coupled monocarboxylate transporters (MCTs) facilitate lactate and proton extrusion, alleviating pH-dependent inhibition of phosphofructokinase 1 (PFK1) to sustain glycolytic abundance (97).

Concomitantly, HIF-1 suppresses mitochondrial fatty acid oxidation by downregulating c-Myc and its targets, medium-chain and long-chain acyl-CoA dehydrogenases(MCAD/LCAD) and inhibiting the PTEN pathway (98, 99). To maintain oxidation equilibrium, HIF-1α activation under hypoxia suppresses mitochondrial respiration by inhibiting oxygen consumption and fatty acid oxidation via HIG2-mediated suppression of lipolysis. It also reprograms glucose and glutamine metabolism, while impairing electron transport chain activity, leading to ROS accumulation and altered energy production (100). This dual mechanism preserves redox homeostasis and ensures lipid availability for membrane biosynthesis. The resulting lactate-rich, acidic TME further reinforces tumor progression via histone lactylation, an epigenetic modification linking metabolic byproducts to transcriptional reprogramming. However, the precise mechanisms underlying lactate-mediated transcriptional regulation and immune suppression remain elusive (101, 102).

Ammonium metabolic reprogramming in prostate cancer constitutes a pathogenic cornerstone that drives tumor initiation, progression, and therapeutic resistance through its dual role as a metabolic integrator and microenvironmental modulator (103). This reprogramming directly responds to coordinated oncogenic alterations—including MYC amplification, androgen receptor signaling hyperactivity, and mTOR pathway activation, which dysregulate core ammonium-metabolizing enzymes as GS, GLS and SLC (104, 105). Concurrently, ammonium accumulation remodels the tumor microenvironment by skewing immune cell differentiation toward immunosuppressive phenotypes and activating cancer-associated fibroblasts (106, 107). Metabolic intermediates including α-ketoglutarate and acetyl-CoA fulfill biosynthetic demands for nucleotide/lipid production while enabling epigenetic reprogramming via TET (Ten-eleven translocation enzymes) dioxygenase-mediated DNA demethylation (108, 109). Furthermore, glutathione synthesized from glutamine-derived precursors confers treatment resistance by scavenging therapy-induced reactive oxygen species, thereby suppressing radiation- and chemotherapy-triggered autophagic cell death and establishing a self-perpetuating oncogenic circuit (110, 111). These metabolic alterations not only intrinsically reprogram tumor cells but also profoundly remodel the TME and associated immune regulation through ammonium metabolic reprogramming.

### 2.3 Ammonium metabolism in the TME: impact on immune cells

The role of ammonium metabolism in tumor immune regulation has drawn significant attention. Research indicates that the metabolic reprogramming of tumor cells leads to the overproduction and accumulation of ammonium, which directly disrupts immune cell functions, inhibiting T cell proliferation and cytotoxicity (112). Moreover, ammonium accumulation alters the metabolic state of immune cells, further weakening their anti-tumor responses (113). These findings highlight ammonia’s critical role in tumor-induced immune suppression and offer a theoretical foundation for developing novel immunotherapy strategies.

Ammonium affects immune cells through several mechanisms. Firstly, the abnormal accumulation of ammonium in immune cells causes metabolic disruptions, impairing the function of T cells, NK cells, and other immune cells. Recent studies highlight that the effectiveness of anti-tumor T cell responses depends on nutrient availability and the metabolic flexibility between cancer cells and immune cells. Tumor cells compete with T cells for essential nutrients in the TME, particularly glucose and amino acids involved in nucleotide synthesis, such as glutamine, glycine, and serine (114–116). These molecules are crucial for both cancer and immune cells to meet biosynthetic and energy demands. Deficiencies or excessive consumption of Arg, Glu, and Branched-chain amino acids (BCAAs) can compromise the ability of tumor-infiltrating lymphocytes (TILs) to clear cancer cells (117). For example, plasma arginine levels have been found to decrease in various cancer types, suggesting that tumors may deplete this amino acid. Arginine depletion via arginase activity can restrict T cell activation and contribute to the establishment of an immunosuppressive environment (118). This is mainly due to the similar amino acid requirements of TILs and cancer cells, with TILs often at a disadvantage in this competition. Arginine plays an essential role in immune responses, particularly in patients with severe trauma, immune suppression, or cancer cachexia, where arginine demand exceeds endogenous production (119–121). Immune cells, particularly CD4+ and CD8+ T cells, depend on sufficient arginine concentrations to maintain effector functions. Arginine synthesis defects, often linked to ASS1 deficiency, are common in cancer cells. Similarly, glutamine is another vital nutrient for both cancer and immune cells. Cancer cells have a high dependence on glutamine, and excess glutamine can stimulate tumor growth while also supporting immune cell function. BCAAs are critical for T cell activation, and their absence impedes T cell expansion and effector differentiation (122).

In the TME, T cell activation is closely associated with ammonium metabolism. Specific mechanisms may involve glutaminase activity promoting effector functions in TH1 cells and cytotoxic CD8+ T cells while inhibiting TH17 cells (123). Additionally, SLC1A5 plays a critical role in the polarization and inflammatory activity of TH1 and TH17 cells (124). Immune checkpoint molecules like PD-1 (programmed death-1) and CTLA-4 (Cytotoxic T lymphocyte-associated antigen-4) function as negative regulators of immune activation, and when triggered by tumor or tumor-associated cells, they suppress immune responses (105, 125–128). A strong relationship exists between checkpoint pathways and cellular metabolism. Immune checkpoint blockade (ICB) therapy can directly influence the metabolism of both immune cells and cancer cells, particularly through the upregulation of PD-1 expression (129). Although CTLA-4 and PD-1 are part of separate pathways, they exert similar inhibitory effects on effector T cell metabolism, including downregulation of AKT phosphorylation, reduced amino acid uptake, and general suppression of metabolic activity. Depletion of tryptophan within the TME can impair anti-tumor immune function of infiltrating lymphocytes. Cells within the TME facilitate immune evasion by reprogramming ammonium metabolism, thereby inhibiting the functions of infiltrating immune cells. Tumor cells, tumor-associated macrophages, and certain dendritic cells can decrease local tryptophan levels by provoking metabolic enzymes, including indoleamine 2,3-dioxygenase (IDO1) and tryptophan 2,3-dioxygenase (TDO2) (130). Increased activity of tryptophan catabolic enzymes promotes the deposition of tryptophan to its metabolite kynurenine (131–133). The depletion of tryptophan in the TME impairs effector T cell function, while kynurenine degradation products can suppress tumor immunity by inducing the generation of Foxp3+ regulatory T cells (Tregs) (134). Recent studies indicate that alanine is crucial for early T cell activation and the re-stimulation of memory CD8+ T cells. However, due to the limited expression of ALT1/2 (or GPT1/2) and low transaminase activity, which restricts alanine biosynthesis, T cells need extracellular alanine for protein synthesis (135). This suggests that during nutrient deprivation, the consumption of extracellular alanine by cancer cells negatively impacts T cell function. The metabolic composition of the TME significantly influences both tumor cells and infiltrating T cells. Glutamine-derived nitrogen is essential for the clonal expansion and differentiation of activated T cells into effector cells (122, 136). However, limiting glutamine consumption through SLC1A5 deficiency or restricted local glutamine availability can promote the expression of Foxp3, a key transcription factor for Treg lineage specification (124, 137). Decreased β-lactam degradation releases α-KG-dependent demethylation at the Foxp3 locus, which promotes the generation of suppressive Tregs and inhibits TH1 differentiation (138). Another study suggests that the accumulation of 2-HG is likely due to increased transamination activity mediated by glutamate Oxaloacetate Transaminase(GOT1), resulting in promoter methylation at the Foxp3 locus and reduced TcB induction (139). Furthermore, glutamine is a precursor for glucosamine synthesis, which is essential for protein glycosylation and has been shown to be critical for activated T cell function (140, 141). Therefore, increased glutamine consumption by cancer cells can modulate anti-tumor immunity by depleting the local glutamine pool required for effector T cell responses, while promoting the development of suppressive Treg populations. A recent research showed that while cancer cells are sensitive to glutamine antagonism, effector T cells can redirect their metabolism towards a more oxidative, long-lived activation phenotype (142). Additionally, ammonium accumulation is closely associated with the redox balance of immune cells, as redox imbalance further hampers their functions, particularly the antigen-presenting capability of dendritic cells (143). By inhibiting dendritic cell maturation from the immature to the mature form, ammonium weakens their ability to initiate T cell responses, thus reducing the immune system’s ability to surveil and combat tumor cells. Furthermore, ammonium alters the types of cytokines secreted by dendritic cells, diminishing their ability to activate T cells and further limiting anti-tumor immune responses (144).

Ammonium TME acidification mainly results from lactate accumulation via anaerobic glycolysis. Ammonium contributes by suppressing aerobic respiration and promoting glycolysis through ammonia-mediated metabolic reprogramming, thereby exacerbating lactate accumulation and acidosis. Reported inhibition of TCA enzymes (e.g., pyruvate dehydrogenase) by ammonia enhances anaerobic glycolysis, further accelerating acidification. Research has shown that ammonium’s inhibitory effects on immune cells, such as T cells and macrophages, are closely associated with TME acidification. This acidification decreases immune cell activity and facilitates tumor cell immune escape. For example, ammonium inhibits perforin maturation, leading to reduced levels of mature perforin in NK cells and consequently diminishing NK cell cytotoxicity. Specifically, ammonium impairs the conversion of perforin from its precursor to the mature form, disrupting its normal function. Perforin maturation and function are pH-dependent within the lysosome. Ammonium raises the lysosomal pH, interfering with perforin maturation. Perforin typically transitions from its precursor to its active form in a low-pH environment. Ammonium accumulation weakens the acidic environment of the lysosome, preventing effective perforin maturation or causing its degradation, thus diminishing its cytotoxic activity. Ammonium not only reduces the level of mature perforin in NK cells but also alters the distribution of other lysosomal markers (e.g., LAMP-1). Ammonium suppresses NK cell cytotoxicity primarily through a dose-dependent reduction in perforin protein levels, without altering its intrinsic activity. This rapid and reversible effect which is independent of transcriptional regulation suggests post-translational impairment of perforin maturation. *In vivo* studies show that mature perforin decreases at 1 mM ammonium and becomes undetectable at 4–5 mM (145). Mechanistically, ammonium disrupts acidic compartments, as indicated by reduced LysoTracker staining, likely impairing the pH-dependent maturation of perforin (79).

**Figure 3:**
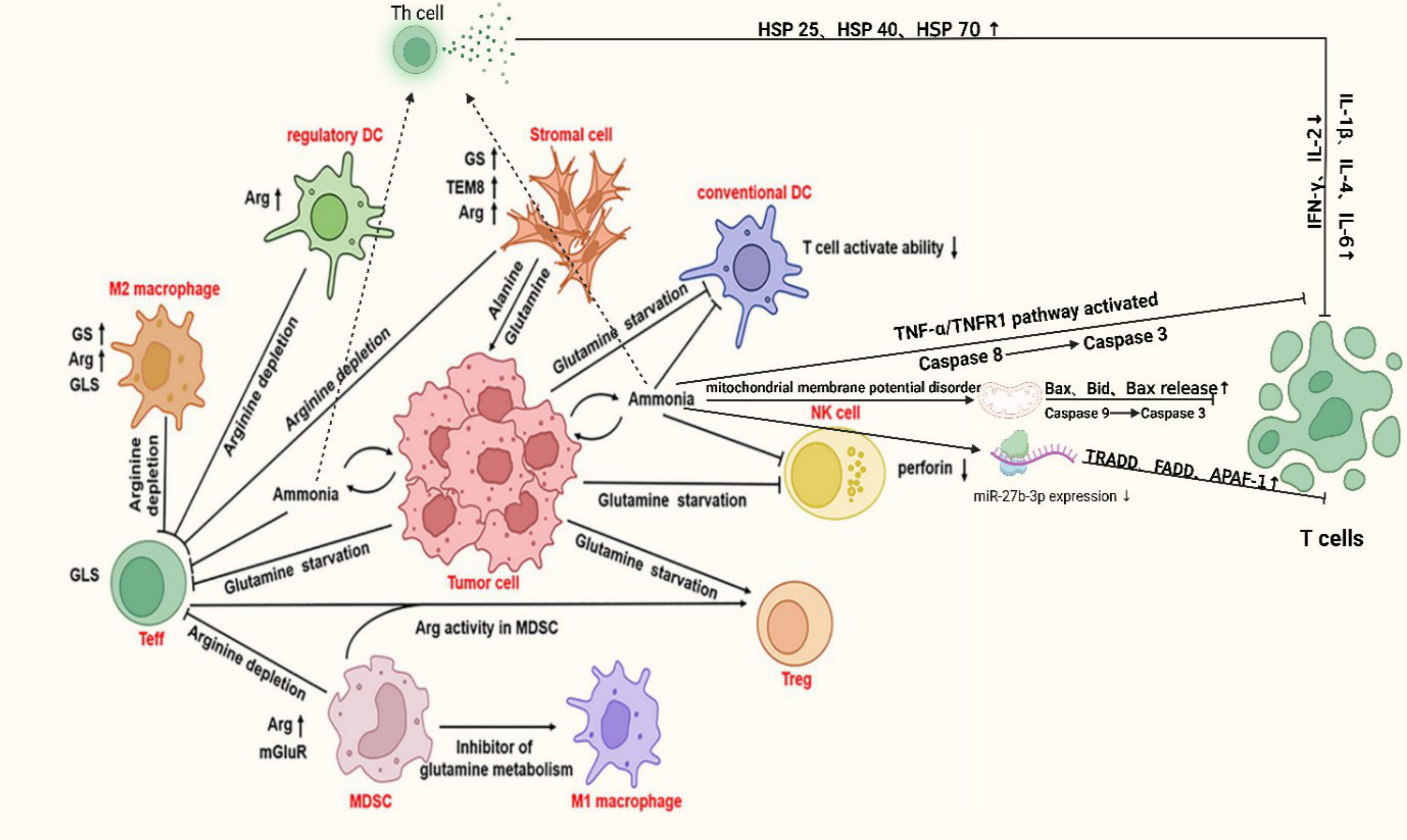
Mechanism of immune cell been suppressed in PCa. In the tumor microenvironment, tumor cells drive immune dysfunction by competitively depleting key metabolites such as glutamine and arginine. Tumor and stromal cells upregulate GS and tumor endothelial marker 8 (TEM8), promoting tumor proliferation and angiogenesis while exhausting extracellular glutamine, thereby impairing T cell activation. M2 macrophages and regulatory dendritic cells enhance anti-inflammatory functions via arginine metabolism, whereas myeloid-derived suppressor cells (MDSCs) suppress immune responses through autophagy and inhibition of glutamine metabolism. Additionally, TNF-α/TNFR1 pathway activation and mitochondrial membrane potential disruption induce apoptosis in immune cells (e.g., NK cells) via Caspase cascades, coupled with reduced perforin levels and dysregulated miR-27b-9p expression. These interconnected mechanisms, metabolic competition, pro-survival adaptations, and apoptotic signaling, collectively establish a highly immunosuppressive niche, enabling tumor immune evasion. DC, dendritic cell; Arg, Arginine; Bax, BCL-2-associated; X, protein; Bid, BH3-interaction domain death agonist; TRADD, Tumor Necrosis factor receptor-associated death domain protein; FADD, Fas-associating protein with a novel death domain; APAF-1, Apoptotic protease activating factor-1; TEM8, tumor endothelial marker 8 Created in https://BioRender.com.

Furthermore, ammonium metabolism suppresses immune cell effector functions, including cytotoxicity, by altering their energy metabolic pathways, thus providing a favorable growth environment for tumor cells. 1.5 ng ug-1 protein ammonium exposure can induce apoptosis in CD8^+^ T cell (146). Ammonium may promote immune cell apoptosis through several mechanisms: 1) Ammonium activates the TNF-α/TNFR1 signaling pathway, triggering the death receptor-mediated apoptosis. The binding of ammonium to TNF-α activates its death domain, recruits TRADD (Tumor Necrosis factor receptor-associated death domain protein) and FADD (Fas-associating protein with a novel death domain) proteins, forms a complex, activates Caspase-8, and subsequently Caspase-3, leading to apoptosis (147). 2) Ammonia induces apoptosis via the mitochondrial pathway. It downregulates mitochondrial membrane potential, causing pro-apoptotic factors such as Bax, Bid, and Bak to bind to the mitochondrial membrane, disrupt it, release Cytc, and activate Caspase-9, which then activates Caspase-3, inducing cell death (148). 3) Ammonium reduces miR-27b-3p expression, enhancing apoptosis-related gene expression (e.g., TRADD, FADD), thus promoting both death receptor and mitochondrial pathway activation (112, 149). 4) Ammonium exposure alters immune cell cytokine secretion, causing an imbalance in immune responses. Specifically, cytokines secreted by Th1 and Treg cells (e.g., IFN-γ, IL-2) decrease, while those from Th2 and Th17 cells (endocrine IL-1β, IL-4, IL-6) increase, suppressing immune function and promoting apoptosis (150). 5) Ammonium exposure activates heat shock proteins (HSPs) like HSP 25, HSP 40, and HSP 70, which regulate immune factor expression and may play a key role in immune cell apoptosis and immune suppression (151, 152). More details can be found in **Figure 3**. While ammonium accumulation-mediated acidification has been experimentally validated (*in vitro* and *in vivo*) to drive immunosuppression in the prostate cancer TME, primarily through impairing T cell and NK cell cytotoxicity and promoting M2 macrophage polarization. Mechanisms of ammonium metabolic reprogramming orchestrates immunosuppression via cytokine networks and stromal crosstalk remain mechanistically unresolved and demand urgent investigation. To harness potential ammonium metabolism reprogramming targets for the treatment of solid tumors, such as prostate cancer, it is essential to investigate the regulatory genes and associated metabolic enzymes involved in the ammonium metabolism reprogramming process.

### 2.4 Molecular regulation of ammonium metabolic reprogramming in tumors

Tumor cells exhibit distinct ammonium metabolism compared to normal cells, characterized by aberrant expression and activity of SLC, metabolic enzymes, and signal transduction pathway rewiring. The SLC family, comprising 455 members across 66 subfamilies, serves as a critical node in tumor ammonium metabolism. Key transporters such as SLC1A5 (glutamine transporter), SLC7A5(LAT1, A heterodimer of SLC7A11 and SLC3A2), and SLC43A1, facilitate glutamine and leucine uptake, activating the mTOR pathway to drive glutaminolysis (153, 154). This process converts glutamine into glutamate, which is further metabolized to α-ketoglutarate for TCA cycle, fueling tumor proliferation (155). SLC6A14 and SLC43A1 are also implicated in leucine transport, with SLC43A1 overexpression correlating with prostate cancer aggressiveness (156–159). Mechanistically, SLC transporters promote glutamine efflux and leucine influx, sustaining mTOR hyperactivation in tumors (160). SLC7A5 expression is regulated by ATF4 (ATF4 is a known transcriptional activator of ASNS expression) under low leucine conditions via the GAAC pathway and by c-Myc through direct promoter binding, while HIF-2α enhances SLC7A5-mediated mTORC1 phosphorylation (161). Integrative omics analyses reveal that MYC orchestrates ammonium metabolism reprogramming in prostate cancer through direct transcriptional control of GLS, with TCGA-PRAD data confirming co-amplification of MYC and GLS (q < 0.001) (162). Parallel mechanisms involve mutant oncogene-mediated dysregulation of metabolic carriers, notably citrin-dependent upregulation of the aspartate-glutamate transporter SLC25A13. These coordinated alterations drive pathological ammonium redistribution, creating a therapeutically exploitable metabolic vulnerability.

Enzyme dysregulation further defines tumor ammonium metabolism. Urea cycle enzymes, such as CPS1, are downregulated in tumors but paradoxically enhance proliferation via S-adenosylmethionine dependent m6A modification of the aspartate transporter SLC1A3, elevating intracellular aspartate (163). GLUL, overexpressed in pancreatic and liver cancers due to c-Myc driven promoter demethylation, supports tumor growth (105). Glutaminase isoforms (GLS1/2), transcriptionally upregulated by c-Myc and hypoxia, catalyze glutamine-to-glutamate conversion, with glutamate dehydrogenase (GLUD) channeling glutamate into α-KG for the TCA cycle and glutathione synthesis, sustaining redox balance and mTOR-driven anabolism. Adaptive expression of urea cycle enzymes ARG1/2, OTC, ASL and regulators like NAT10, which stabilizes ATF4 mRNA via ac4C modification to upregulate asparagine synthetase (ASNS) optimizing nitrogen utilization for tumor proliferation (164). Collectively, these alterations highlight ammonium’s dual role as a metabolic byproduct and biosynthetic precursor, offering therapeutic targets to disrupt tumor metabolic plasticity (as shown in **Figure 2**).

Current research on targeted therapy for prostate cancer primarily focuses on identifying potential inhibitory mechanisms. Massive researches have concentrated on SLC family, with SLC7A11, the SLC3 family, and SLC7A5 emerging as promising therapeutic targets (165). SLC7A11 activity may be influenced by environmental metal elements such as antimony, which modulate the Nrf2/SLC7A11/GPX4 axis and inhibit ferroptosis in PCa cells (166). The SLC3 family has been implicated in epithelial–mesenchymal transition (EMT) and cell cycle–related apoptosis, while SLC7A5 is speculated to be associated with distant metastasis (167, 168). Several inhibitors targeting SLC transporters are currently in preclinical trials. In addition to transporters, enzymes involved in ammonium metabolism have also drawn attention as potential therapeutic targets. Current research primarily focuses on blocking glutamine utilization in cancer cells using GLS inhibitors such as CB-839 and JHU083. Some of these agents have already progressed to phase III clinical trials, providing new hope for improved therapeutic strategies against prostate cancer.

## 3 Metabolic reprogramming of ammonium metabolism in prostate cancer microenvironment

Metabolic characteristics, metabolic reprogramming, immune microenvironment, and immune evasion are intricately interconnected, forming a complex network that drives the initiation and progression of prostate cancer. Under high concentration of ammonium, prostate cancer cell stays active and keep cell viability via several mechanism. Recent studies show that aspartate levels are elevated in the prostate cancer TME, suggesting partial activation of the urea cycle (169). Higher CPS enzyme activity in prostate cancer tissues compared to controls indicates that the active urea cycle helps mitigate ammonia toxicity. Additionally, excessive urea cycle activation promotes CPS expression, converting ammonia-derived nitrogen into pyrimidine metabolites, supporting tumor cell proliferation (170). Modulating ammonium metabolism holds significant potential in the treatment of prostate cancer, offering deeper insights into its biological characteristics and providing novel therapeutic strategies aimed at improving survival and quality of life for patients with advanced prostate cancer.

### 3.1 Metabolic crosstalk in prostate cancer microenvironment: acidification and its role in immunosuppression TME

The TME in PCa is a complex and dynamic system that plays a critical role in tumor progression, immune evasion, and therapy resistance. Immunosuppressive factors such as HIF-1α, CD73, and PGE2 (Prostaglandin E), along with immunosuppressive cell populations including tumor-associated macrophages (TAMs), regulatory T cells (Tregs), and myeloid-derived suppressor cells (MDSCs), have been shown to be present in the prostate cancer immune microenvironment and may contribute to immune evasion (171–175). TAMs and Tregs are pivotal in shaping the immunosuppressive landscape. TAMs promote immune evasion by secreting immunosuppressive cytokines, including TGF-β and IL-10, which inhibit the function of cytotoxic T cells (particularly CD8+ T cells) and impair antigen presentation. Tregs further contribute to immune suppression by dampening the effect of T-cell responses, thereby enabling tumor cells to evade immune surveillance (176).

**Figure 4:**
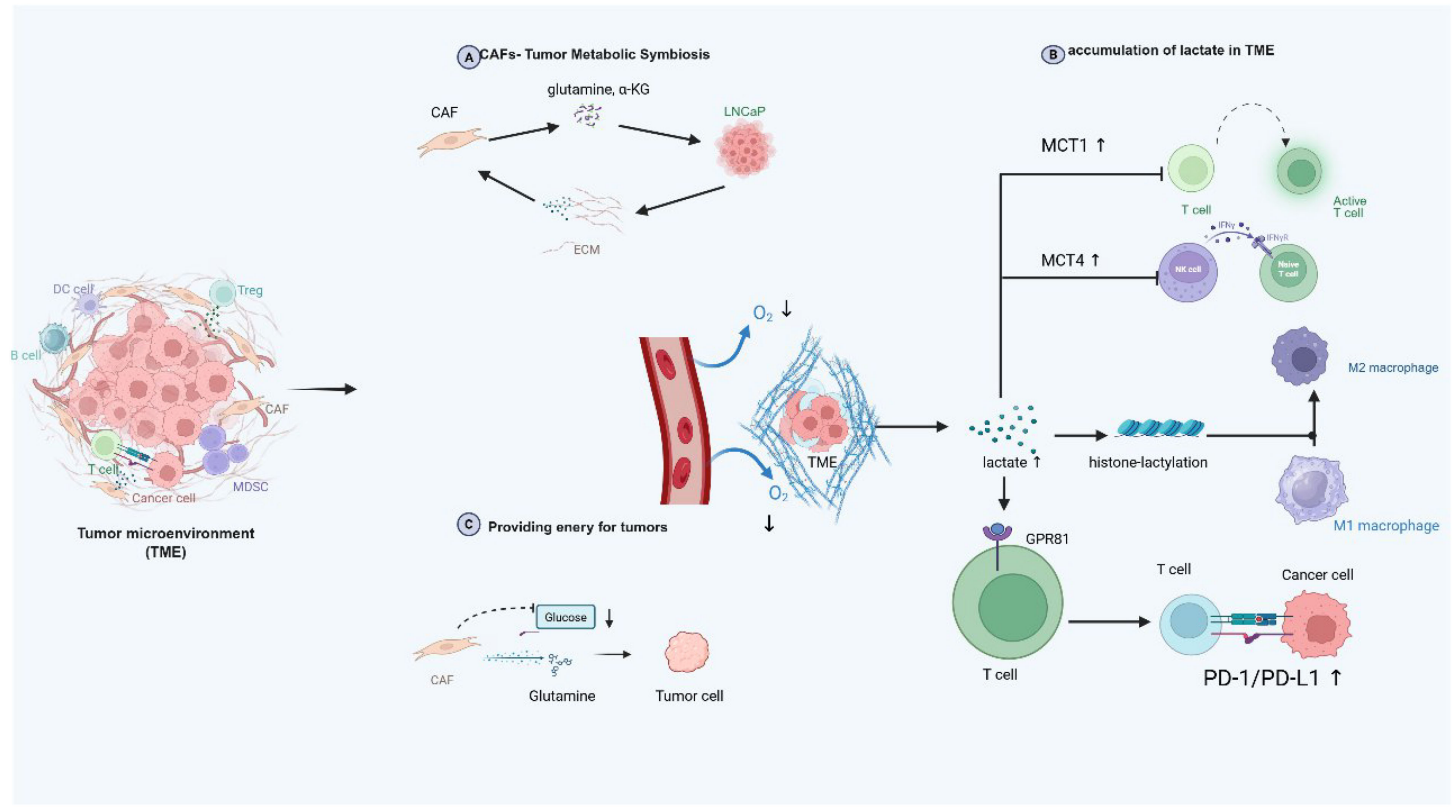
Ammonium metabolism in TME and possible interplay with TME components. This diagram vividly depicts the intricate dynamics within the TME. It showcases the metabolic symbiosis between cancer - associated fibroblasts (CAFs) and tumor cells like LNCaP, where CAFs secrete glutamine and α-ketoglutarate for tumor cells, with reciprocal exchanges via the extracellular matrix. In the oxygen-deprived TME, tumor cells generate lactate, which accumulates and triggers the upregulation of MCT1 and MCT4 transporters. This lactate not only induces histone-lactylation to potentially modify gene expression but also exerts a profound impact on immune cells, such as inhibiting the function of T and NK cells, skewing macrophage polarization towards the M2 phenotype, and engaging with GPR81 on T cells to upregulate the PD-1/PD-L1 pathway for immune evasion. Additionally, CAFs utilize glucose and glutamine, potentially funneling metabolites to fuel tumor cell growth, highlighting the complex interplay of metabolism and immune regulation in the TME that supports tumor progression. TME, tumor microenvironment; CAF, cancer-associated fibroblast; MCT, monocarboxylate transporter; GPR81, G protein-coupled receptor 81, PD-1, programmed death-1, PD-L1, programmed death ligand 1.

Hypoxia alters the metabolic profiles of immune cells, impairing their anti-tumor functions and facilitating immune evasion. Tumor cells exploit these metabolic changes to suppress immune cell activity, for example, by increasing lactate production and accumulating immunosuppressive metabolites, which further inhibit T cell proliferation and cytotoxicity (177, 178). Previous studies have established that metabolic reprogramming in solid tumors like prostate cancer promotes immunosuppression through TME acidification (179). Elevated lactate concentrations (>20 mM) impair immune cell function by competitively inhibiting monocarboxylate transporters (MCTs), MCT1 in T cells and MCT4 in NK cells, leading to metabolic paralysis or apoptosis (180) (**Figure 4**). Simultaneously, lactate drives histone lactylation (Kla), a novel epigenetic modification identified via HPLC-MS/MS analyses, revealing 28 conserved Kla sites. This modification directly reprograms gene expression, exemplified by its capacity to polarize macrophages toward M2 phenotypes through non-inflammatory pathways (181). Notably, M2 macrophages exhibit superior survival under acidic pH compared to M1 counterparts (6). Furthermore, lactate activates oncogenic signaling via G-protein-coupled receptor GPR81, upregulating immune checkpoints like PD-L1 (182, 183). While ammonium metabolic rewiring likely exacerbates TME acidification through glycolytic-lipogenic crosstalk, further *in vivo*/*in vitro* validation is required to delineate these indirect mechanisms. (**Figure 4**).

Beyond ammonia-driven TME acidification that broadly suppresses antitumor immunity, competitive depletion of arginine, a key ammonium metabolism related substrate, represents an underappreciated axis of immune evasion in prostate cancer (184). Androgen signaling as evidenced in castration therapy studies, manipulates this immunosuppressive network by inhibiting arginine metabolism via two synergistic pathways: 1) Myeloid-derived suppressor cells (e.g., CD11b^+^ cells) overexpressing ARG1 deplete extracellular L-arginine, crippling T-cell function through CD3ζ chain downregulation, impaired antigen recognition, and dual activation of the GCN2-eIF2α stress pathway with concurrent mTOR suppression (185–187). 2) Nitric oxide (NO) generated by nitric oxide synthase (NOS) disrupts IL-2 signaling via inhibition of JAK-STAT, ERK, and AKT phosphorylation, destabilizing IL-2 and inducing T-cell apoptosis (188–191). Critically, arginine scarcity triggers a pathological shift in NOS activity, from NO production to superoxide (O_2_^-^) and reactive nitrogen oxide species (RNOS) generation, which amplifies T-cell suppression through combined oxidative damage and signal blockade (192, 193). These mechanisms are exacerbated in castration-resistant prostate cancer (CRPC), where TAMs exhibit elevated ARG I/II activity and heightened susceptibility to low arginine environments. Targeting this metabolic-immune crosstalk may thus yield novel immunotherapeutic strategies for CRPC.

Recent studies reveal that prostate TME regulates tumor progression by modulating ammonium metabolism substrates. *In vitro* studies demonstrate that cancer-associated fibroblasts (CAFs) support hormone-sensitive prostate cancer (HSPC) proliferation via glutamine and α-KG secretion, fueling energy and biosynthetic demands (194, 195). This metabolic symbiosis exhibits biphasic regulation: LNCaP cells upregulate Gln catabolic pathways (e.g., GLS1-dependent glutaminolysis), while CAFs activate extracellular matrix (ECM) remodeling pathways. Notably, GLS1 expression serves as a dynamic biomarker of this metabolic interplay. Broader solid tumor studies corroborate that pharmacological inhibition of CAF-mediated Gln synthesis disrupts tumor metabolic fitness, suggesting combinatorial targeting of tumoral and stromal glutamine metabolism, particularly through dual GLS1/ECM pathway inhibition, may offer promising therapeutic avenues for advanced prostate cancer (196).

### 3.2 New insight of cellular programmed death associated with metabolic reprogram in PCa

Emerging research underscores the intricate crosstalk between metabolic reprogramming and regulated cell death pathways in PCa, where hypoxia and nutrient deprivation within the TME drive adaptive survival mechanisms (197). PCa cells and stromal components sustain a hypermetabolic state characterized by heightened dependence on glutamine and mTORC1-mediated suppression of autophagy, a process critical for oncogenesis, as evidenced by dysregulated autophagy-related genes (e.g., STK11/LKB1) upstream of mTORC signaling (198). The tumor suppressor STK11/LKB1 activates AMPK, a central metabolic sensor that integrates energy stress signals through cross-talk with PI3K, mTOR, and MAPK pathways (199). In LKB1-deficient non-small cell lung cancer (NSCLC) models, AMPK inactivation derepresses CPS1, whose silencing induces nucleotide pool imbalance (reduced pyrimidine/purine ratio), S-phase arrest, and DNA damage, mechanisms hypothesized to operate in PCa, where ammonium metabolism inhibitors (e.g., targeting glutaminase) combined with autophagy modulators (e.g., ULK1 activators) could exploit metabolic vulnerabilities targeting tumor cells (200). Concurrently, ammonium metabolites exhibit mitochondrial toxicity, collapsing membrane potential, and depleting ATP, while nutrient stress activates AMPK to phosphorylate ULK1, initiating pro-survival autophagy (201, 202).

Paradoxically, phosphoserine phosphatase suppresses hepatocellular carcinoma autophagy via the AMPK/mTOR/ULK1 axis, analogous regulatory networks remain uncharacterized in PCa.

**Figure 5:**
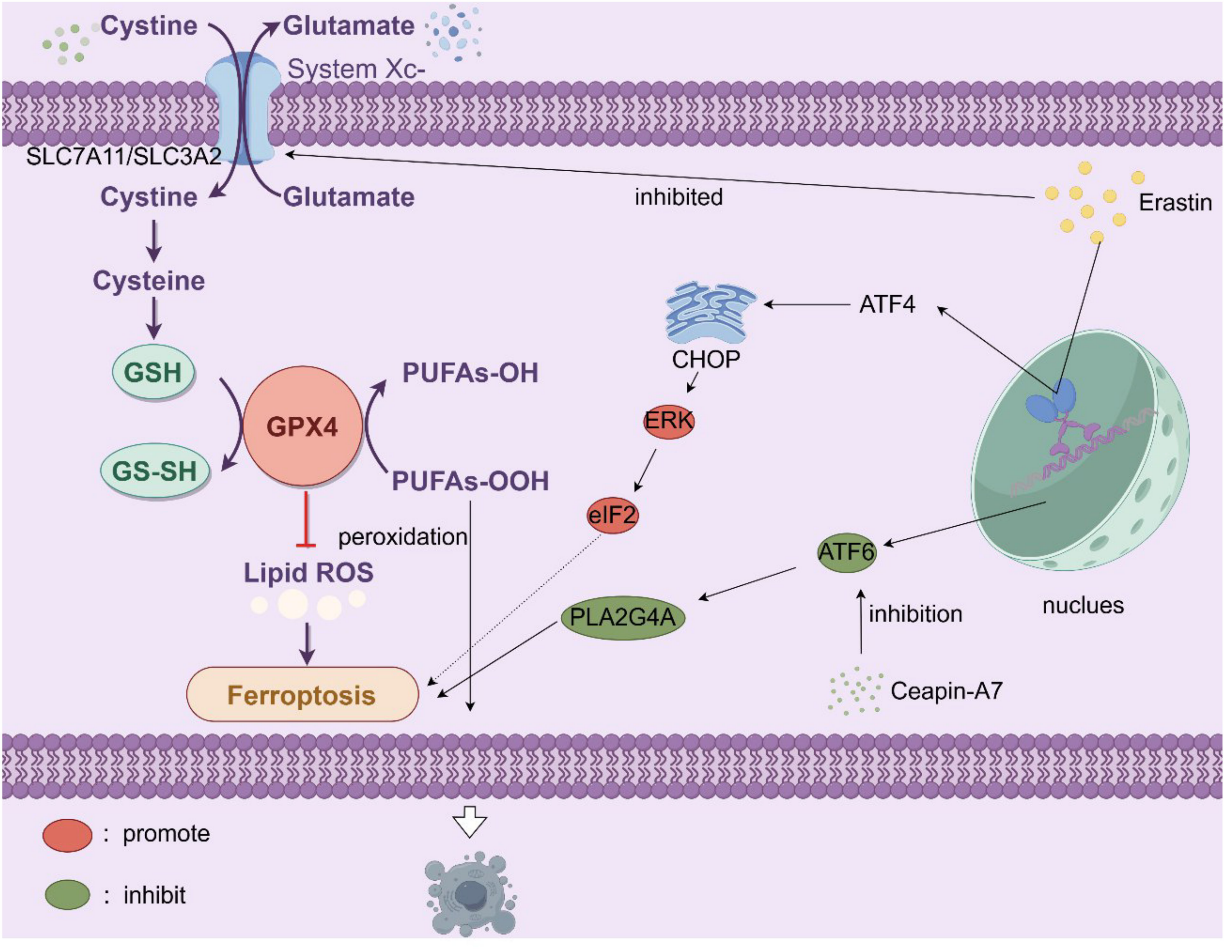
The cell membrane System Xc^-^ (SLC7A11/SLC3A2) facilitates the exchange of extracellular cystine for intracellular glutamate. Cystine is subsequently converted to cysteine, a key component in the synthesis of GSH. GSH is vital as it supplies the reducing power for GPX4 to convert PUFAs-OOH into PUFAs-OH, thereby curbing lipid reactive oxygen species (lipid ROS) accumulation and preventing ferroptosis. However, the compound erastin inhibits System Xc^-^, diminishing cystine uptake and consequently reducing GSH levels. This undermines GPX4’s function, allowing lipid ROS to build up and triggering ferroptosis. Additionally, stress-related signaling pathways play a role. Activating transcription factor 4 (ATF4) is upregulated under stress, leading to the induction of C/EBP homologous protein (CHOP), which in turn activates extracellular signal-regulated kinase (ERK), promoting ferroptosis. Activating transcription factor 6 (ATF6), whose inhibition can be mediated by Ceapin-A7, influences the activity of phospholipase A2 group IVA (PLA2G4A), which can contribute to lipid peroxidation and the onset of ferroptosis. In essence, disruptions in cystine-glutamate transport, antioxidant defenses, and activation of specific signaling cascades all converge to initiate ferroptosis, characterized by iron-dependent lipid peroxidation. Erastin, ferroptosis activator; GPX4, glutathione peroxidase 4; PUFA, polyunsaturated fatty acid; ATF, activating transcription factor, C/EBP, CCAAT/enhancer-binding protein; ERK, extracellular signal-regulated kinase; PLA2G4A, phospholipase A2 group IVA.

PCa also engages ferroptosis, an iron-dependent death pathway driven by glutathione peroxidase 4 (GPX4) downregulation and lipid peroxidation (202). Key regulators include SLC7A11 and SLC3A2 (LAT 1), components of the cystine/glutamate antiporter system Xc-. Erastin-induced inhibition of system Xc- depletes glutathione (GSH), disrupting redox balance and triggering ferroptosis (203, 204). Intriguingly, ferroptosis inducers activate non-canonical endoplasmic reticulum (ER) stress cascades, including the ATF4-CHOP/ERK-eIF2 axis, while bypassing apoptosis, a resilience mechanism attenuated in LNCaP-AI cells with elevated ATF6 expression. ATF6-mediated transcriptional activation of PLA2G4A confers ferroptosis resistance, whereas pharmacological inhibition of ATF6 (e.g., Ceapin-A7) synergizes with enzalutamide to suppress CRPC growth (205). Detailed mechanisms are portrayed in **Figure 5**. These findings implicate ATF transcription factors as critical nodes linking ammonium metabolic rewiring (e.g., glutaminolysis) to ferroptosis sensitivity. Collectively, co-targeting metabolic dependencies (e.g., glutaminase inhibitors, ammonium scavengers) and death evasion mechanisms (e.g., ferroptosis inducers, ATF6 inhibitors) may overcome therapeutic resistance, with preclinical validation urgently needed for strategies combining autophagy modulation (AMPK/mTOR/ULK1 targeting), ferroptosis potentiation (system Xc− blockade), and stromal disruption (CAF-derived glutamine inhibition) (205, 206). Unresolved questions include the tissue-specific regulation of ULK1 complexes in PCa autophagy, role of CPS1 in nucleotide metabolism, and spatiotemporal dynamics of ATF6-mediated therapy resistance, all critical for advancing precision therapies in this metabolic-death nexus.

## 4 Application prospects of ammonium metabolism in prostate cancer

### 4.1 Key therapeutic targets in ammonium metabolism for prostate cancer

Ammonium metabolism involves key enzymes that regulate metabolic processes, including ASNS, GS, and GLS. By modulating these enzymes, the concentrations of ammonium metabolism substrates and products are altered, which in turn affects PCa behaviors such as proliferation, differentiation, and invasion (207). Specific enzymes and their targeted drugs, along with clinical trial information, are outlined in the **Table 2**.

**Table 2:** researches focus on the utilization of ammonia metabolism inhibitor.

Therapeutic target	Drug	Drug number	Cancer	Trial type	Trial number	Stage	Material/participants	Reference
GLS	Teleglenastat	CB-839	mCRPC	phase II	NCT04824937	UNKNOWN	30	(290)
	Teleglenastat	CB-839	solid tumor	phase I	NCT02071862	COMPLETED	210	(291)
	Teleglenastat	CB-839	solid tumor	PHASE1|PHASE2	NCT03965845	COMPLETED	53	(292)
	Teleglenastat	CB-839	solid tumor	PHASE1|PHASE2	NCT03875313	TERMINATED	33	(293)
	DON prodrug	JHU083	PCa	pre-clinical trials		COMPLETED	PC-3 cell line	(227)
Arginase	Arginase Inhibitor	INCB001158	solid tumor	phaseIb	NCT03910530	COMPLETED	18	(293)
	Recombinant Human Arginase 1	PEG-BCT- 100	CRPC	phase I	NCT02285101	COMPLETED	22	(293)
ASNS	Sirpiglenastat	DRP-104	solid tumors	PHASE1|PHASE2	NCT04471415	TERMINATED	61	(294)
	DON prodrug	DRP-104	lymphoma	pre-clinical trials		COMPLETED	mice	(228)
GS	Vitamin C		PCa	pre-clinical trials		COMPLETED	PC-3 cell line	(295)
SLC40A1	Leonurine		PCa	pre-clinical trials		COMPLETED	PC-3 cell line	(214)
SLC1A5	ASCT2 antibody		solid tumor	pre-clinical trials		COMPLETED	SK-Hep1	(296)

These studies focus on prostate cancer or include solid tumors for ammonium metabolism inhibition treatment in prostate cancer. Detailed efficacy results and study information can be found on the respective study websites, which encompass data from prostate cancer patients, prostate cancer cell lines, and animal models of prostate cancer. This form primarily compiles data from clinical trials listed on https://clinicaltrials.gov and from public databases such as PubMed prior to the clinical experiments.

These drugs show significant promise for metabolic therapy in prostate cancer. This section highlights new research demonstrating that CB-839, a drug that targets GLS, inhibits prostate cancer proliferation through several mechanisms (90). GLS inhibition prevents the conversion of glutamine to glutamate in cancer cells (208). This process generates α-ketoglutarate, a key fuel for the TCA cycle, and inhibiting this step deprives cancer cells of energy for proliferation. Furthermore, glutamate not only serves as an essential TCA cycle intermediate but also helps maintain the cellular redox balance (209, 210). In cancer cells, GLS supports the production of glutamate, which is vital for the synthesis of GSH, a major antioxidant that scavenges ROS, triggering oxidative stress and eventually leading to apoptosis (211). Another mechanism involves GLS inhibition mediated activation of apoptosis pathways in prostate cancer cells, evidenced by the upregulation of apoptosis-related genes. This process may involve the p53 pathway (a known tumor suppressor) or the activation of other proapoptotic genes, resulting in programmed cell death (212). Moreover, GLS inhibition impairs the metabolic adaptability of cancer cell, potentially triggering excessive autophagy, resulting in apoptosis. Inhibition of other ammonium metabolism enzymes works through similar mechanisms, halting cancer cell proliferation via the coupling of ammonium metabolism to the TCA cycle. Research on ASNS reveals that inhibiting ASNS in mCRPC may reduce resistance to androgen therapy (19). ASNS also helps cancer cells survive through anti-apoptotic mechanisms. In addition, ASNS inhibition promotes aspartate accumulation, activates p53, and suppresses prostate cancer growth by regulating metabolic pathways, DNA repair, cell cycle progression, and apoptosis (22). Combining CB-839, a well-tolerated GLS inhibitor, with ASNase effectively limits asparagine synthesis and inhibits the growth of CRPC tumors driven by TP53 mutations. These findings open a new therapeutic avenue for prostate cancer and other solid tumors. Inhibition of arginase suppresses prostate cancer cells, as these cells often rely on external arginine for rapid proliferation. Arginine deprivation thus becomes an important therapeutic target (213). Additionally, arginine depletion inhibits T cell proliferation and promotes the recruitment of TAMs and myeloid-derived suppressor cells (MDSCs), facilitating immune evasion. Nitric oxide (NO) signaling plays dual roles in tumor immune evasion and tumorigenesis. Arginine depletion reduces NO synthesis and may alter its protumor and antitumor effects.

New research has focused on the SLC transporter family, with findings showing that SLC proteins facilitate the transport of substances such as ammonium and glutamine across lipid bilayers. One study revealed that leopurine inhibits SLC40A1, reducing its transport efficiency (214). Furthermore, SLC40A1 expression is linked to microRNA-18a-5P, a noncoding RNA that can regulate gene expression by binding to specific genes. In this context, SLC40A1 inhibition leads to the binding of microRNA-18a-5P with RUNX1, affecting the transcription process and influencing prostate cancer cell proliferation.

### 4.2 Ammonium metabolism-related biomarkers in prostate cancer

Prostate specific antigen (PSA) has been the gold standard for diagnosing PCa in hospitals and research institutes (215). However, while PSA has high sensitivity, its specificity is low, and factors such as prostate inflammation or other noncancerous diseases can also lead to elevated PSA levels (216–218). Furthermore, improvements such as PSA velocity, PSA density, and the free-to-total PSA ratio have shown only marginal improvements in specificity. Consequently, new biomarkers are urgently needed to aid in the early detection and treatment of PCa. Biochemical changes in cancer cells occur earlier than cytological, imaging, or functional changes. Metabolic profiling of bodily fluids is a promising method for identifying clinically valuable non-invasive biomarkers. A study revealed significantly elevated concentrations of Asp, Tyr, Val, Arg, Cit, Gly, Gln, and His (P < 0.05) in the blood samples of PCa patients, whereas Glu, Trp, Orn, and Ser levels were reduced. Among these, the Glu/Gln ratio is the most valuable for research, with specific AUC, sensitivity, and specificity data provided in the table (219). Metabolic changes in urinary vesicles may also serve as potential diagnostic biomarkers (220). Recent studies have shown that specific amino acids in these vesicles can accurately differentiate PCa from BPH and classify PCa into different stages, as detailed in the **Table 3**.

**Table 3:** New metabolic markers of prostate cancer related to ammonium metabolism.

Metabolite	Test platform	Sample	AUC	P value	Direction in cancer
Glu/Gln	LC-MS/MS	Blood serum	0.98	<0.05	Glu/Gln=8.1
Arginine	MS	Blood serum	0.67	0.039	↑
Kynurenine	MS	Blood serum	0.72	0.009	↑
Valine	MRS	Blood serum	0.69	0.032	↑
Gln+Glu	MRS	Blood serum	0.7	0.031	↑
Glutamate	MRS	Blood serum	0.66	0.049	↑
Pyruvate	MRS	Blood serum	0.71	0.015	↑
Lysine	MRS	Blood serum	0.71	0.015	↑
Histidine	MRS	Blood serum	0.69	0.024	↑
Tyrosine	MRS	Blood serum	0.67	0.037	↑
Phenylalanine	MRS	Blood serum	0.73	0.011	↑
Sarcosine	FA	Blood serum	0.67	0.03	↑
Spermine	UPLC–MS/MS	Pre-biopsy urine	0.83	<0.001	↓

This table summarizes the ammonium metabolic markers that may be associated with the development of prostate cancer during ammonium metabolism. These novel markers offer greater accessibility compared to PSA. Specifically, the table details their measurement methods along with the area under the curve (AUC) and P-values. MRS, Magnetic Resonance Spectroscopy; LC-MS/MS, Liquid Chromatography-Tandem Mass Spectrometry; MS, Mass Spectrum; FA, fluorometric assay in serum; UPLC, Ultra Performance Liquid Chromatography.

### 4.3 Ammonium metabolism-related treatment combined with other therapies

#### 4.3.1 Combining ammonium metabolism inhibition with targeted therapy

TP53-targeted therapy combined with metabolism inhibitors exploits the metabolic vulnerability caused by TP53 deletions, particularly the reliance on the asparagine synthesis pathway. Recent *in vitro* experiments have shown that tumor cells lacking TP53 produce a large amount of aspartic acid when cultured *in vitro (* 22). TP53 deletion activates the ATF4/ASNS pathway, which enhances *de novo* synthesis of asparagine and supports tumor cell survival and proliferation in androgen-deprived or nutrient-limited environments. ATF4, a transcriptional activator of ASNS, is upregulated when TP53 deleted, leading to increased ASNS expression and increased intracellular asparagine synthesis (22).

GLS1 (GAC, glutaminase C), such as CB-839 and DONs, suppress glutamine metabolism, mainly by affecting GDH in the active site of GAC reducing its conversion to glutamate and thus limiting the synthesis of asparagine. Since asparagine synthesis depends on glutamate as a precursor, GLS inhibitors further suppress asparagine synthesis by reducing glutamine availability. Combining GLS inhibitors with targeted therapy can suppress glutamine production and asparagine synthesis via their respective metabolic pathways, restoring tumor cell sensitivity to ASNase and significantly reducing asparagine levels, thereby increasing therapeutic efficacy (221).

This combination therapy not only effectively disrupts the metabolic adaptation caused by TP53 mutations but also provides a new direction for the treatment of CRPC and other prostate cancers by targeting key metabolic pathways. The combination of GLS inhibitors and ASNase has shown promising therapeutic potential against TP53-mutated CRPC tumors in experimental studies and may offer new approaches for the personalized treatment of these cancers. Further treatment strategies are needed to confirm which targets GLS should be combined with for PCa treatment.

#### 4.3.2 Combining ammonium metabolism targeting with chemotherapy

Paclitaxel resistance in mCRPC is associated with multiple cellular and metabolic mechanisms (222). Resistant cells often exhibit increased invasiveness, motility, and tumorigenic potential, along with metabolic reprogramming. Specifically, there is an increase in oxidative phosphorylation (OXPHOS), GSH synthesis, and reactive oxygen species (ROS) scavenging, which are closely linked to glutamine metabolism. Gln metabolism supports OXPHOS by providing energy, promotes GSH synthesis, and enhances ROS scavenging, helping to counteract paclitaxel-induced oxidative stress (223). Additionally, paclitaxel-resistant PCa cells often upregulate the expression of the antiapoptotic protein Bcl-2 and increase GSH levels, further enhancing antioxidant defense and resistance.

Furthermore, Gln metabolism contributes to chemotherapy resistance in prostate cancer cells by modulating drug efflux, particularly through the upregulation of the ATP-binding cassette transporter ABCB1 (224). ABCB1 reduces the intracellular concentration of chemotherapy drugs, thereby diminishing their therapeutic efficacy (225, 226). The activation of other pro-survival signaling pathways, such as the PI3K/Akt, MAPK, and NF-kB pathways, as well as the regulation of DNA repair mechanisms (e.g., ATM/ATR and Chk1/Chk2 pathways), also play critical roles in paclitaxel resistance. Therefore, Gln metabolism is essential for the cellular energy supply and aids tumor cells in adapting to paclitaxel treatment stress by regulating redox balance and drug efflux, thereby providing a survival advantage for resistance.

#### 4.3.3 Integration of ammonium metabolism inhibition with immune checkpoint blockade

The inhibition of glutamine utilization by DON (Gln antagonist) and its prodrugs, such as JHU 083 and DRP-104, has been shown to enhance antitumor immune responses (142, 227, 228), suggesting a potential strategy when combined with immune checkpoint inhibitors. DRP-104 has been tested in the NCT 04471415 clinical trial to assess its preliminary safety and efficacy as a monotherapy or in combination with anti-PD-L1 immunotherapy (atezolizumab) in patients with advanced solid tumors such as PCa. However, this study remains unfinished, and the results have not yet been made available.

DON is considered a promising anticancer agent, but its clinical application has been limited by dose-limiting toxicity. DON exerts its effects by inhibiting several enzymes that utilize glutamine in both tumor and normal tissues mainly digestive tract. To minimize toxicity to normal tissues, DRP-104, a peptide prodrug of DON, was developed to preferentially convert DON in tumor tissues. Studies have demonstrated that DON and its prodrugs, such as JHU 083 and DRP-104, enhance antitumor immune responses by inhibiting glutamine utilization and that their combination with immune checkpoint inhibitors could improve therapeutic outcomes. Furthermore, DRP-104 effectively inhibits the carbon and nitrogen glutamine pathways in CRPC cells, thereby suppressing their growth. Currently, DRP-104 is being evaluated int he NCT 04471415 clinical trial for its safety and efficacy as a monotherapy or in combination with anti-PD-L1 immunotherapy (atezolizumab) in patients with advanced solid tumors, however, the study was recently terminated, and the results has not yet been released.

#### 4.3.4 Synergistic combinations of ammonium metabolism inhibitors

CB-839, when combined with ASNS, reduces asparagine levels. Studies have shown that this combination effectively limits the synthesis of asparagine and inhibits the growth of CRPC tumors induced by TP53 mutations. This strategy offers a new therapeutic approach for solid tumors, such as prostate cancer. The combination of GLS inhibitors, such as CB-839, with ASNase, works synergistically by not only inhibiting asparagine synthesis but also enhancing its degradation. CB-839 inhibits the metabolic conversion of glutamine, thereby reducing asparagine synthesis and increasing tumor cell dependency on asparagine. ASNase further degrades asparagine, limiting the availability of essential amino acids to cancer cells and thereby inhibiting their growth. This combination therapy has demonstrated promising therapeutic potential in prostate cancer and other types of tumors. Moreover, while the use of ASNS alone can cause adverse effects such as pancreatitis and thrombosis, its combination with CB-839 effectively mitigates these risks (19).

#### 4.3.5 Combination of ammonium metabolism drugs and radiotherapy

Excessive ROS production leads to an imbalance between ROS and the cell’s antioxidant defense, resulting in DNA damage, a key mechanism in radiation therapy-mediated tumor treatment (40). Radio-resistance in prostate cancer is mediated by enhanced glutamine metabolism, whose downstream product GSH confers therapeutic resistance. Targeting glutamine metabolism suppresses GSH synthesis and restores radiosensitivity, offering a promising therapeutic strategy. Additionally, GLS inhibition modifies the cellular redox state, altering the cell’s response to radiation therapy. Cancer stem cells (CSCs) constitute a unique population within tumors with self-renewal and differentiation potential. These cells are more resistant to radiation and chemotherapy than regular cancer cells and are typically located in the core of tumors, making them difficult to eliminate completely with conventional treatments. Studies have shown that glutamine metabolism is crucial not only for the energy and nitrogen supply of cells but also for the maintenance of CSCs. Glutamine regulates CSCs through signaling pathways such as the mTOR and Notch. In this study, GLS inhibition reduced glutamine metabolism in prostate cancer cells, significantly decreasing the expression of stem cell markers (such as ALDH, OCT4, and Sox2), with the cells exhibiting reduced stemness characteristics. By decreasing the stemness of cancer cells, GLS inhibition decreases their resistance to radiation, thus enhancing the radiosensitivity of prostate cancer.

Chemical inhibition of key points of glutamine metabolism, such as GLS and MYC, or genetic knockdown of these genes and glutamine transporters, suppresses glutamine decomposition, thereby inhibiting crucial CSC-driving pathways (WNT/β-catenin, the oxidative stress response, and the DNA damage response), resulting in CSC depletion both *in vitro* and *in vivo* and enhancing tumor radiosensitivity. ALDH-positive (ALDH+) PCa CSC populations exhibit radio-resistance, tumor initiation, and metastatic traits (229–231). Inhibition of GLS with BPTES or genetic silencing of GLS/GLS2 genes increases radiation sensitivity in lung and prostate cancer cell lines.

#### 4.3.6 Combination of ammonium metabolism and endocrine therapy

Prostate cancer cells adapt to extreme conditions, such as hypoxia in the TME, through metabolic reprogramming to sustain growth and proliferation, as discussed earlier. Specifically, androgen treatment induces excessive oxidative stress in prostate cancer cells, leading to cell death. To maintain rapid proliferation, these cells require efficient protein synthesis and amino acid metabolism (232). This may lead to the accumulation of ammonium, which in turn activates intracellular antioxidant responses and other metabolic pathways, alleviating the oxidative stress induced by endocrine therapy. Ammonium accumulation enhances the cellular antioxidant capacity by activating a series of enzymes and pathways, such as glutamine synthetase, leading to the production of antioxidant compounds such as GSH.

Additionally, recent studies have shown that the transporter SLC1A5 (ASCT-2), which is involved in glutamine metabolism is regulated by androgens (233). In prostate tumor samples, ASCT-2 expression is significantly higher than that in normal prostate tissue, especially in castration-resistant tumors, and is associated with the growth and metastatic potential of prostate cancer cells. Prostate cancer cells regulate glutamine metabolism through ASCT2 (SLC1A5) and LAT1/3 transporters, which support amino acid uptake for proliferation and metastasis. ASCT2 is highly expressed in prostate cancer cells, facilitating glutamine uptake, cell cycle progression, mTORC1 activation, fatty acid synthesis, and energy metabolism. Inhibition of ASCT2 significantly reduces glutamine uptake, cell proliferation, tumor growth, and metastasis by lowering E2F pathway proteins. Glutamine metabolism in prostate cancer is also regulated by oncogenes like MYC, androgen receptors (AR), and mTOR, with metabolic tracing studies highlighting glutamine’s role in energy and precursor synthesis (234). Targeting ASCT2 and glutamine metabolism is a potential therapeutic strategy for prostate cancer (235). DU-145 cells, in particular, show marked glutamine dependency, with prominent GLS gene and protein expression. GLS inhibition (e.g., via BPTES) significantly reduces cell viability, demonstrating potent antitumor effects, especially in AR-independent cell lines (29, 236, 237). A combined therapy targeting both AR and glutamine metabolism could be more advantageous. In conclusion, AR plays a crucial role in PCa progression by regulating the glutamine metabolism network. Although AR-targeted therapies have been effective in the early stages, the emergence of CRPC is associated with adaptive changes, including increased glutamine utilization. Advanced PCa cells shift towards glutamine metabolism to meet energy demands, positioning it as a central hub in the PCa metabolic network. Therefore, targeting glutamine metabolism is a promising strategy, particularly since PCa cells (including CSCs) are highly dependent on glutamine during disease progression. Combining therapies targeting both AR and glutamine metabolism may play a greater role in combating advanced PCa and overcoming treatment resistance.

Metformin, an antidiabetic agent, promotes glutamine metabolic dependency in PCa cells by restricting glucose-derived carbon entry into the TCA cycle, thereby forcing PCa cells to utilize glutamine reductive carboxylation for citrate production. Pharmacological inhibition of GLS via CB-839 or BPTES amplifies PCa cell sensitivity to metformin (29). Compared with single-agent approaches, combined glutamine depletion and metformin treatment synergistically increase radiosensitivity in autophagy-deficient PCa models (40). These findings indicate a synergistic interaction between glutamine metabolism suppression and metformin in PCa cells.

#### 4.3.7 Limitations of drugs targeting ammonium metabolism

Although systematic investigations into the adverse effects of ammonium metabolism-targeting agents in prostate cancer remain limited, their toxicological profiles have been relatively well characterized in other solid tumors, such as breast, pancreatic, and gastrointestinal cancers (211, 222, 238). Representative glutamine metabolism inhibitors, including DON, CB-839, and BPTES, have exhibited varying degrees of toxicity during development, with gastrointestinal side effects being particularly prominent and constituting a major barrier to clinical translation (239–242). These adverse events are likely due to the broad inhibition of multiple glutamine metabolism-related enzymes. Additional toxicities such as hypocalcemia and reversible myelosuppression have also been reported (243–245). In terms of pharmacological properties, DON and BPTES show moderate potency, poor metabolic stability, low water solubility, and limited selectivity for GLS, resulting in inferior antitumor efficacy compared to CB-839 (246, 247). By contrast, CB-839 is a highly selective GLS inhibitor that has demonstrated promising therapeutic efficacy across multiple tumor models. Several studies have identified potential biomarkers predictive of CB-839 sensitivity, including the intracellular glutamate/glutamine ratio, expression levels of the GLS isoform GAC, and GLS enzymatic activity (90, 93, 248, 249). These indicators could facilitate the development of predictive tools and support personalized treatment strategies, although further validation is warranted.

Nevertheless, both intrinsic and acquired resistance to CB-839 remains a critical challenge for widespread application. Tumor cells can bypass glutamine metabolism by activating alternative pathways; for instance, the upregulation of pyruvate carboxylation to generate oxaloacetate (OAA) has been observed in breast and pancreatic ductal adenocarcinomas (250). Furthermore, fatty acid oxidation (FAO) is markedly upregulated in resistant tumor cells, providing additional substrates such as acetyl-CoA and propionyl-CoA to fuel the TCA cycle via citrate synthase and succinyl-CoA synthesis (251, 252). Interestingly, the mTORC1 signaling pathway plays a pivotal role in this adaptive metabolic reprogramming, especially under glutamine-restricted conditions. Notably, combinatorial treatment with CB-839 and mTOR inhibitors has shown synergistic effects in preclinical studies, offering a compelling rationale for dual-targeted strategies and highlighting their potential for future clinical translation (253, 254).

## 5 Future outlook and direction

The metabolic reprogramming of prostate cancer cells has garnered significant attention, and drugs targeting metabolic processes are constantly being developed. However, there studies have notable limitations. Focusing solely on the metabolism of prostate cancer cells is insufficient, as the TME operates as a dynamic and balanced system. The interactions among immune cells, cytokine networks, and ammonium metabolism within the TME still requires further investigation. Additionally, the role of ammonium metabolism in prostate cancer remains underexplored, with insufficient experimental validation of its underlying mechanisms. Only by elucidating the upstream and downstream regulatory mechanisms can new targeted therapies be developed. Additionally, we have identified varying degrees of interference in experimental studies on ammonia metabolic reprogramming. As pointed out by De Berardinis et al., the changes in tumor ammonia metabolism between *in vivo* and *in vitro* experiments may differ significantly (255). For example, in *in vitro* studies, shRNA is often used to silence the expression of metabolic enzymes to observe their effects on ammonia metabolism, whereas *in vivo*, the affected sites may be more numerous and complex. This discrepancy suggests a potential direction for future research: whether the conclusions regarding the impact of ammonium metabolism on tumor progression are reliable. Furthermore, relying solely on metabolic flux is insufficient to determine the complete picture of ammonia metabolic reprogramming in tumor cells and its relative contribution compared to other metabolic pathways. Therefore, the most suitable approach currently is to provide a snapshot of metabolism rather than a reliable measurement of metabolic flux. More sensitive methods, such as using hyperpolarized 13C-labeled probes, may ultimately support *in vivo* flux analysis (256).

### 5.1 Potential effects of ammonium metabolism on various components in the TME of prostate cancer

As integral components of the TME, tumor-infiltrating immune cells (TIICs) can significantly alter the immune landscape of tumors (25, 257–259). Previous studies have demonstrated that the interactions between TIICs and prostate cancer cells can serve as therapeutic targets, with experimental evidence supporting these relationships (260, 261). However, immune checkpoint-based therapies have not yielded satisfactory outcomes in prostate cancer patients. Consequently, the impact of metabolic reprogramming, particularly ammonium metabolism, on TIICs within the TME warrants further investigation.

Recent studies have highlighted the detrimental effects of ammonium metabolism on nonspecific immune cells within the TME, particularly NK cells. Ammonium accumulation disrupts NK cell metabolism by impairing glycolysis and OXPHOS, both of which are essential for ATP production and cytotoxic activity (142, 227). Ammonium accumulation specifically inhibits hexokinase and lactate dehydrogenase in glycolysis, while sparing other enzymes (262). It may modulate TCA cycle activity via secondary messengers like cAMP, though further validation is needed (263). Additionally, ammonium impairs mitochondrial function, further inhibiting OXPHOS and NK cell activation (142). In addition to energy metabolism, ammonium accumulation affects lipid metabolism, altering membrane fluidity and receptor activation, which compromises NK cell efficiency in target cell recognition and elimination. Moreover, ammonium contributes to metabolic acidosis in the TME by forming ammonium ions (NH_4_^+^), lowering the pH, disrupting redox balance, and further suppressing NK cell function. These studies suggest that ammonium accumulation may induce programmed cell death of immune cells within TME, such as NK cells, through oxidative imbalance and mitochondria-mediated autophagy, thereby promoting immune evasion by prostate cancer cells. However, the specific mechanisms underlying this process remain to be elucidated. Moreover, additional therapeutic targets within this pathway are yet to be identified, and combination strategies involving immune checkpoint blockade (ICB) may offer enhanced therapeutic efficacy. This approach has demonstrated efficacy in the treatment of other solid tumors. For instance, the V9032 inhibitor has been shown to enhance tumor cell sensitivity to PD-1-targeted therapy in breast cancer (223, 264).

Ammonium also promotes tumor immune escape by modulating immune regulatory pathways. It enhances the immunosuppressive activity of Tregs, promoting their expansion and increasing the secretion of TGF-β and IL-10, which dampens antitumor immunity. Additionally, ammonium upregulates immune checkpoint molecules such as PD-L1 on tumor cells, directly inhibiting NK cell activation. By altering metabolic pathways, including glycolysis and fatty acid oxidation, ammonium promotes immune tolerance and weakens NK cell effector functions, particularly in solid tumors such as PCa. Macrophages, especially those in the M1 and M2 phenotypes, play crucial roles in tumor progression, particularly in PCa (265). M1 macrophages are involved in antitumor immunity, whereas M2 macrophages promote tumor growth, metastasis, and immune suppression. M2 macrophage presence is linked to worse clinical outcomes in several cancers, including PCa, and may cooperate with Tregs to create an immunosuppressive environment that assists the tumor to evade immune surveillance. Targeting M2 macrophages and their pathways, in addition to regulating Tregs, could offer a potential strategy for treating lethal PCa.

Matrix of TME are also influenced by the consumption of glutamine leading to a decrease in energy supply required for T cells to perform immune functions, thereby suppressing their activity (266). Arginine plays a vital role in T cell immunity by synthesizing nitric oxide, and its deficiency inhibits T cell proliferation and function. Tryptophan metabolism via the IDO enzyme pathway suppresses T-cell proliferation and promotes immune tolerance. Nonessential amino acids produced during amino acid metabolism are critical for the metabolic reprogramming of T cells. After activation, T-cells shift from glycolysis to amino acid synthesis and utilization to support proliferation and differentiation, which competes with tumor cells, leading to T-cell suppression. The uptake of glutamine depends on amino acid transporters such as ASCT2, which play a key role in T cell differentiation. Arginine enters T cells via the CAT-1 transporter, and its deficiency impacts T-cell proliferation, differentiation, and cytokine secretion. Asparagine aids in protein synthesis and restores mTOR activity, helping tumor cells proliferate and maintaining T-cell function (267, 268). Joint deprivation of arginine and asparagine leads to extensive T-cell death, indicating their complementary role in maintaining T-cell activity. Additionally, serine synthesis is essential for T-cell proliferation and tumor growth. Limiting serine suppresses T-cell proliferation, although some tumors adapt by increasing phosphoglycerate dehydrogenase (PHGDH) activity. Although, targeting PHGDH may inhibit tumor growth, but its impact on the immune response remains unclear.

According to previous studies, ammonium metabolism not only affects TIICs in the TME but also influences cytokines secreted by immune cells or other immunoreactive substances upstream of genes. These noncellular components may serve as potential targets for future drug therapies and can function as prognostic indicators. Recent research has demonstrated that pH levels in the TME significantly impact immune cell function, cytokine secretion, and ammonium metabolism reprogramming, which may contribute to the decreased pH value in the TME (269). A novel immunoconjugate compound, L-DOS47, was utilized to increase the pH of an acidic medium *in vitro* to evaluate its effects on Jurkat, a human T lymphoblast-like cell line.

### 5.2 Influence of ammonium metabolism on other major therapeutic modalities

Current prostate cancer treatments primarily include surgical and nonsurgical approaches. Among nonsurgical treatments, castration is currently the most attractive option; however, in androgen-insensitive prostate cancers such as CRPC, androgen therapy may be ineffective (232, 270, 271). The long-term use of ADT can lead to metabolic disorders in patients. Drugs that target metabolic reprogramming may synergize with ADT to some extent. Metabolic reprogramming, as an emerging research direction, has shown considerable efficacy in combination with other treatments in various solid tumors. However, in prostate cancer treatment, particularly regarding the combined treatment of ammonium metabolism and androgens, effective studies remain scarce, with most focusing on glycolysis. Ammonium metabolism is also a primary metabolic mode after tumor cell metabolic reprogramming, potentially representing a future research direction. Previous studies have demonstrated that AR signaling (DHT, 5-alpha-dihydrotestosterone) primarily enhances glutamine (Gln) uptake by regulating the expression of various Gln transporters, such as SLC1A4 (ASCT1), SLC1A5 (ASCT2), SLC3A2 (4F2hc), SLC7A5 (LAT1), and SLC43A1 (LAT3), to meet the proliferative and differentiative demands of PCa cells (39, 233, 236). ADT alone typically induces the development of CRPC, during which CRPC cells often undergo metabolic reprogramming toward enhanced ammonium metabolism, characterized by a higher Gln utilization rate. This metabolic shift in CRPC is reflected in increased Gln uptake and upregulation of key regulatory genes involved in glutaminolysis (272–274). The elevated demand for Gln is further evidenced by the overexpression of Gln transporters such as ASCT2 and LAT1, as well as the increased expression of the androgen-independent GLS isoform GAC. As PCa progresses to more aggressive stages, both PCa cells and prostate cancer CSCs exhibit Gln addiction (274, 275). Therefore, targeting the Gln metabolic network represents a unique opportunity to overcome therapeutic resistance and sensitize tumor cells to anticancer treatments, potentially offering greater benefit than AR-targeted monotherapy (39, 168). CRPC cells generally rely on glutamine metabolism for energy supply, providing a mechanistic explanation for the effectiveness of ammonium metabolism inhibitors, especially glutamine metabolism inhibitors, in combination with second-generation androgen receptor antagonists (276). Moreover, the AR signaling axis can activate GLS activity, promoting the conversion of glutamine to glutamate and providing energy substrates for the tricarboxylic acid cycle, another potential mechanism for the use of metabolic inhibitors in combination with ADT. Further studies comparing LNCaP cells (androgen-sensitive prostate cancer) with mCRPC cells revealed a stronger metabolic dependence on glutamine in LNCaP cells. The androgen signaling pathway not only drives prostate cancer proliferation and survival but also provides metabolic advantages through ammonium metabolic reprogramming. Combined targeting of AR signaling and glutamine metabolic pathways holds promise as a new strategy to improve CRPC treatment. Advanced metabolomics techniques can assist in accurately screening precise metabolic targets to optimize treatment. Although ADT is effective, the metabolic reprogramming it induces drives CRPC development. Therapies incorporating metabolic targets, such as those inhabit amino acid metabolism, are expected to significantly improve outcomes in CRPC patients.

In addition to developing therapeutic agents that target the reprogramming of ammonium metabolism in prostate cancer, it is essential to identify reliable indicators for evaluating treatment efficacy, such as sensitivity-related gene signatures and metabolomic profiling (277). While previous imaging techniques have mainly focused on magnetic resonance spectroscopy (MRS), which detects localized concentrations of metabolites such as choline, citrate, and various amino acids, these metabolic profiles reflect tumor metabolic intensity and may indicate responsiveness to ammonium-targeted therapies (278, 279). When integrated with conventional imaging and biopsy, MRS can aid in treatment assessment; however, its clinical application remains limited due to the low concentration of metabolites relative to water, necessitating high-field magnets, long acquisition times, and specialized expertise in spectral analysis. To overcome these limitations, novel metabolic PET tracers have been developed, offering improved sensitivity and specificity in detecting early metabolic changes in prostate cancer (280–282). Additionally, advanced hyperpolarized ^13^C-labeled spectral MRI techniques provide dynamic and real-time evaluation of tumor metabolism (283). Metabolomics-based research may further reveal valuable biomarkers for therapy monitoring, as the reprogramming of ammonium metabolism in prostate cancer generates a range of intermediate metabolites, such as polyamines, citrate, and various amino acids, as well as key metabolic enzymes, which hold promise as indicators of therapeutic response (284). Further experimental validation and clinical investigation are needed to establish their clinical utility.

## 6 Conclusion

Overall, ammonium metabolism, particularly downstream glutamine metabolism, is crucial for the metabolic treatment of prostate cancer. Corresponding metabolic inhibitors can be targeted, and products from this metabolic pathway can serve as diagnostic or prognostic markers for prostate cancer. Additionally, ammonium metabolism inhibitors can enhance immune cell function by affecting the immune microenvironment in prostate cancer, achieving therapeutic effects. Therefore, the role of ammonium metabolism in the immune microenvironment of prostate cancer remains worth exploring, along with its potential application value in diagnosis and treatment.
